# The Burden of Parasitic Zoonoses in Nepal: A Systematic Review

**DOI:** 10.1371/journal.pntd.0002634

**Published:** 2014-01-02

**Authors:** Brecht Devleesschauwer, Anita Ale, Paul Torgerson, Nicolas Praet, Charline Maertens de Noordhout, Basu Dev Pandey, Sher Bahadur Pun, Rob Lake, Jozef Vercruysse, Durga Datt Joshi, Arie H. Havelaar, Luc Duchateau, Pierre Dorny, Niko Speybroeck

**Affiliations:** 1 Department of Virology, Parasitology and Immunology, Faculty of Veterinary Medicine, Ghent University, Merelbeke, Belgium; 2 Institute of Health and Society (IRSS), Faculty of Public Health, *Université catholique de Louvain,* Brussels, Belgium; 3 National Zoonoses and Food Hygiene Research Center, Kathmandu, Nepal; 4 Section of Epidemiology, Vetsuisse Faculty, University of Zürich, Zürich, Switzerland; 5 Department of Biomedical Sciences, Institute of Tropical Medicine, Antwerp, Belgium; 6 Everest International Clinic and Research Center, Kathmandu, Nepal; 7 Clinical Research Unit, Sukraraj Tropical and Infectious Disease Hospital, Kathmandu, Nepal; 8 Institute of Environmental Science and Research, Christchurch, New Zealand; 9 Centre for Zoonoses and Environmental Microbiology, National Institute for Public Health and the Environment (RIVM), Bilthoven, The Netherlands; 10 Institute for Risk Assessment Sciences, Faculty of Veterinary Medicine, Utrecht University, Utrecht, The Netherlands; 11 Department of Comparative Physiology and Biometrics, Faculty of Veterinary Medicine, Ghent University, Merelbeke, Belgium; University of Oklahoma Health Sciences Center, United States of America

## Abstract

**Background:**

Parasitic zoonoses (PZs) pose a significant but often neglected threat to public health, especially in developing countries. In order to obtain a better understanding of their health impact, summary measures of population health may be calculated, such as the Disability-Adjusted Life Year (DALY). However, the data required to calculate such measures are often not readily available for these diseases, which may lead to a vicious circle of under-recognition and under-funding.

**Methodology:**

We examined the burden of PZs in Nepal through a systematic review of online and offline data sources. PZs were classified qualitatively according to endemicity, and where possible a quantitative burden assessment was conducted in terms of the annual number of incident cases, deaths and DALYs.

**Principal Findings:**

Between 2000 and 2012, the highest annual burden was imposed by neurocysticercosis and congenital toxoplasmosis (14,268 DALYs [95% Credibility Interval (CrI): 5450–27,694] and 9255 DALYs [95% CrI: 6135–13,292], respectively), followed by cystic echinococcosis (251 DALYs [95% CrI: 105–458]). Nepal is probably endemic for trichinellosis, toxocarosis, diphyllobothriosis, foodborne trematodosis, taeniosis, and zoonotic intestinal helminthic and protozoal infections, but insufficient data were available to quantify their health impact. Sporadic cases of alveolar echinococcosis, angiostrongylosis, capillariosis, dirofilariosis, gnathostomosis, sparganosis and cutaneous leishmaniosis may occur.

**Conclusions/Significance:**

In settings with limited surveillance capacity, it is possible to quantify the health impact of PZs and other neglected diseases, thereby interrupting the vicious circle of neglect. In Nepal, we found that several PZs are endemic and are imposing a significant burden to public health, higher than that of malaria, and comparable to that of HIV/AIDS. However, several critical data gaps remain. Enhanced surveillance for the endemic PZs identified in this study would enable additional burden estimates, and a more complete picture of the impact of these diseases.

## Author Summary

Various parasites that infect humans require animals in some stage of their life cycle. Infection with these so-called zoonotic parasites may vary from asymptomatic carriership to long-term morbidity and even death. Although data are still scarce, it is clear that parasitic zoonoses (PZs) present a significant burden for public health, particularly in poor and marginalized communities. So far, however, there has been relatively little attention to this group of diseases, causing various PZs to be labeled neglected tropical diseases. In this study, the authors reviewed a large variety of data sources to study the relevance and importance of PZs in Nepal. It was found that a large number of PZs are present in Nepal and are imposing an impact higher than that of malaria and comparable to that of HIV/AIDS. These results therefore suggest that PZs deserve greater attention and more intensive surveillance. Furthermore, this study has shown that even in settings with limited surveillance capacity, it is possible to quantify the impact of neglected diseases and, consequently, to break the vicious circle of neglect.

## Introduction

Various parasites infecting humans depend on vertebrate animals to complete their life cycle. Humans most commonly become infected with these zoonotic parasites through consumption of infected hosts or through fecal-oral contamination. The results of these infections may vary from asymptomatic carriership to long-term morbidity and even death. Although data are still scarce, it is clear that these parasitic zoonoses (PZs) present a significant burden for public health, particularly in poor and marginalized communities [1], [2]. Moreover, PZs can lead to significant economic losses, both directly, through their adverse effects on human and animal health, and indirectly, through control measures required in the food production chain [3], [4].

Estimates of the impact of diseases on public health, generally referred to as burden of disease, may be valuable inputs for decision makers when setting policy priorities and monitoring intervention programs. In Nepal, it is now recognized that health sector needs should be prioritized, and that disease burden should be considered as one of the bases for this prioritization [5]. However, disease burden estimates are not readily available. While the World Health Organization and the Global Burden of Disease (GBD) initiative have generated such estimates for Nepal, these were largely based on regional extrapolations, and, more importantly, included only a limited number of PZs [6], [7]. If disease burden estimates are to be used for priority setting, an incomplete assessment of the burden of PZs may lead to a vicious circle of under-recognition, a wrong ranking of priorities and under-funding for research, prevention and control programs [8].

To address this issue, a disease burden assessment of PZs was conducted in Nepal. Ideally, the primary data sources for such studies would be official surveillance data and death registers. In Nepal, however, these data sources have limited value in terms of PZs. The official passive surveillance system of the Government of Nepal, the *Health Management Information System* (HMIS), has been reported to suffer from inconsistencies, incomplete reporting, and under-reporting from mainly central-level and private hospitals [9], [10]. Active surveillance systems are in place, but only target certain vaccine-preventable diseases, and not PZs. Death registration is reported to have a completeness rate of 32% [11]. We therefore opted for a more comprehensive approach, based on a systematic review of all possible secondary data sources related to PZs in Nepal from 1990 to 2012. This comprehensive review allowed us to identify endemic and possibly endemic PZs, and, subsequently, to quantify the disease burden of those PZs for which sufficient quantitative data were available.

## Materials and Methods

The main objective of this study was to provide a comprehensive overview of the public health impact of PZs in Nepal. To this end, a step-wise approach was taken:

1. Systematic review of national and international peer-reviewed and grey literature;
2. Qualitative assessment: classification of considered PZs according to (presumed) endemicity status and data availability; and
3. Quantitative assessment: quantification of health impact of endemic PZs in terms of the annual number of cases, deaths and Disability-Adjusted Life Years (DALYs), for the year 2006.

### Considered PZs

The twenty PZs considered in this study are listed in Table 1. This selection is based on a recent review of the world-wide socioeconomic burden of PZs [1] and a review of emerging food-borne parasites [12], as many PZs may be classified as being food-borne. Seven of the considered PZs also belong to the group of neglected tropical diseases, i.e., leishmaniosis, cystic and alveolar echinococcosis, cysticercosis, food-borne trematodosis, schistosomosis and soil-transmitted helminthosis [13], [14].

**Table 1:** Parasitic zoonoses considered in the Nepalese burden of disease study (in alphabetical order).

Parasitic zoonosis	Involved species	Transmission route(s)*
Alveolar echinococcosis	*Echinococcus multilocularis*	Fecal-oral
Angiostrongylosis	*Angiostrongylus cantonensis*	Snail-borne (meat-borne, fecal-oral)
*Anisakidae* infections	*Anisakis* spp., *Pseudoterranova* spp.	Fish-borne
Capillariosis	*Capillaria philippinensis*	Fish-borne
	*Capillaria hepatica*	Meat-borne
	*Capillaria aerophila*	Fecal-oral (earthworm-borne)
Cystic echinococcosis	*Echinococcus granulosus*	Fecal-oral
Cysticercosis	*Taenia solium*	Fecal-oral
Dirofilariosis	*Dirofilaria* spp.	Arthropod-borne
Diphyllobothriosis	*Diphyllobothrium latum*	Fish-borne
Foodborne trematodoses	*Fasciola* spp.; *Fasciolopsis buski*	Plant-borne
	*Opisthorchis* spp.; *Clonorchis sinensis*	Fish-borne
	*Paragonimus* spp.	Arthropod-borne
	Intestinal flukes	Various
Gnathostomosis	*Gnathostoma* spp.	Amphibian/reptile-borne
Sparganosis	*Spirometra* spp.	Amphibian/reptile-borne
Taeniosis	*Taenia* spp.	Meat-borne
Toxocarosis	*Toxocara* spp.	Fecal-oral (meat-borne)
Toxoplasmosis	*Toxoplasma gondii*	Fecal-oral, meat-borne
Trichinellosis	*Trichinella* spp.	Meat-borne
Zoonotic intestinal helminth infection	*Ascaris suum*; *Trichuris* spp.	Fecal-oral
	*Ancylostoma* spp.; *Strongyloides stercoralis*	Fecal-oral, transcutaneous
Zoonotic intestinal protozoal infection	*Giardia duodenalis*; *Cryptosporidium* spp.; *Blastocystis* spp.	Fecal-oral
	*Sarcocystis* spp.	Meat-borne
Zoonotic leishmaniosis	*Leishmania* spp. (excluding *L. donovani*)	Arthropod-borne
Zoonotic schistosomosis	*Schistosoma japonicum*	Water-borne
Zoonotic trypanosomosis	*Trypanosoma cruzi*	Arthropod-borne

* *Less common transmission routes are shown in parentheses.*

### Systematic review

**Figure 1:**
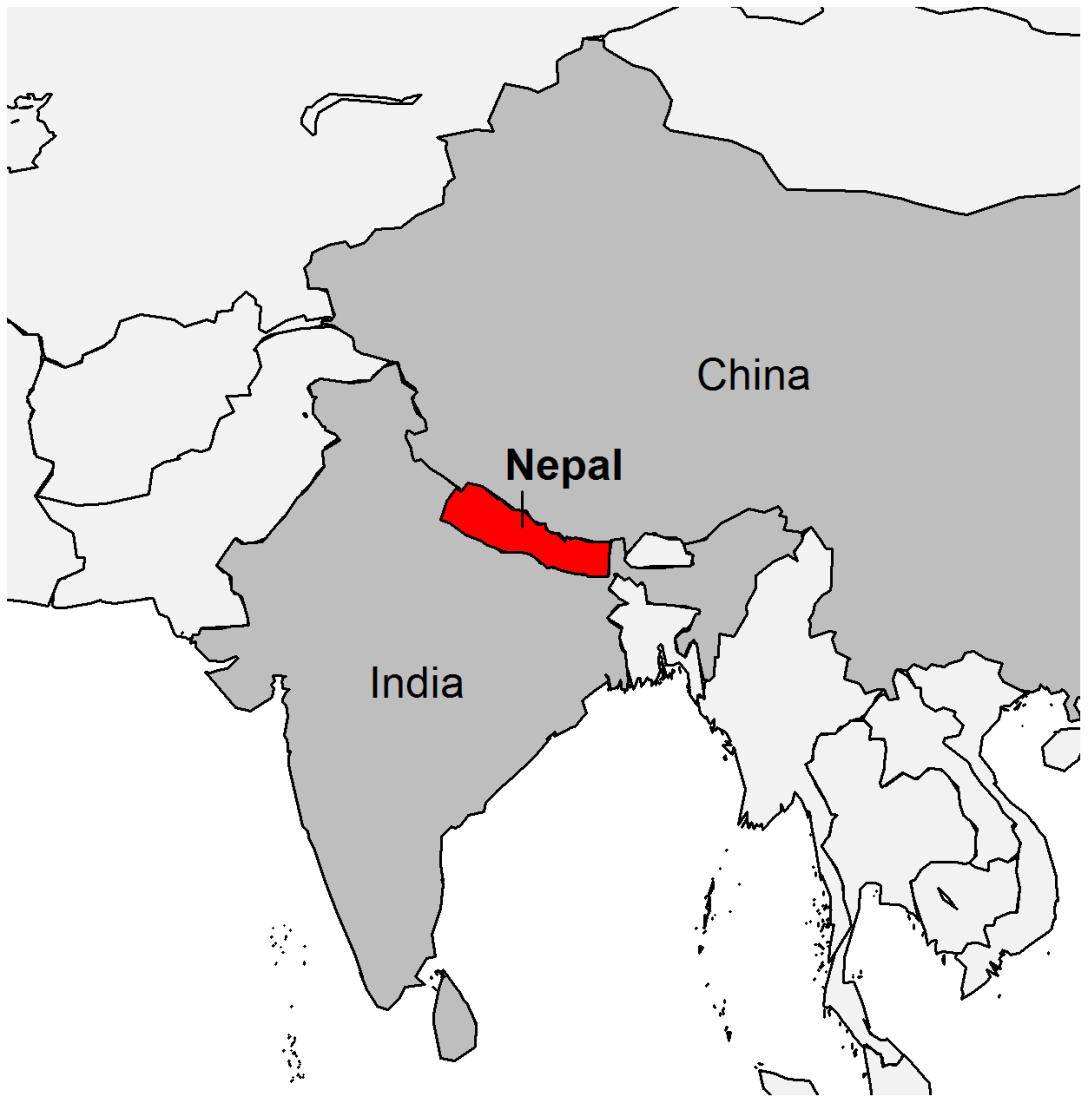
Nepal, in red, bordered by India in the south, and China in the north.

Direct and indirect evidence on the occurrence of the considered PZs was located through a systematic search of national and international peer-reviewed and grey literature. Direct evidence was defined as any data on prevalence, incidence or mortality of the PZ in humans. Indirect evidence was defined as occurrence of the concerned parasite in animal hosts or in the environment (e.g., water, soil). If no direct or indirect evidence could be identified from Nepal (further referred to as “local” evidence), recent case reports were sought from (North) India, Nepal's largest neighbor with whom it shares an open border in the west, south and east, and from the Tibet Autonomous Region, which borders Nepal in the north (Figure 1).

For each PZ, we constructed a search phrase consisting of the key word “Nepal” and any element of a list containing the name of the PZ, possible synonyms, and the name(s) of the causative parasite(s) (Table S1-1 in Supporting Information S1). Manuscript titles were retrieved through searching PubMed, Web of Science, WHO Global Health Library, Asia Journals OnLine (AsiaJOL) and MedInd. If available, the major Nepalese journals were additionally searched through their websites (Table S1-2 in Supporting Information S1). In addition, the thesis libraries of Tribhuvan University (Kathmandu, Nepal) and the Institute of Animal Agriculture Sciences (Rampur, Chitwan district) were manually explored to find relevant manuscripts. Dissertations were also collected from the website of the Veterinary Public Health master course jointly organized by Chiang Mai University (Thailand) and Freie Universität Berlin (Germany), as this program has a regular intake of Nepalese students. No dissertations were sought from countries neighboring Nepal, as we did not have prior knowledge of masters courses organized in these countries with a regular intake of Nepalese students.

**Figure 2:**
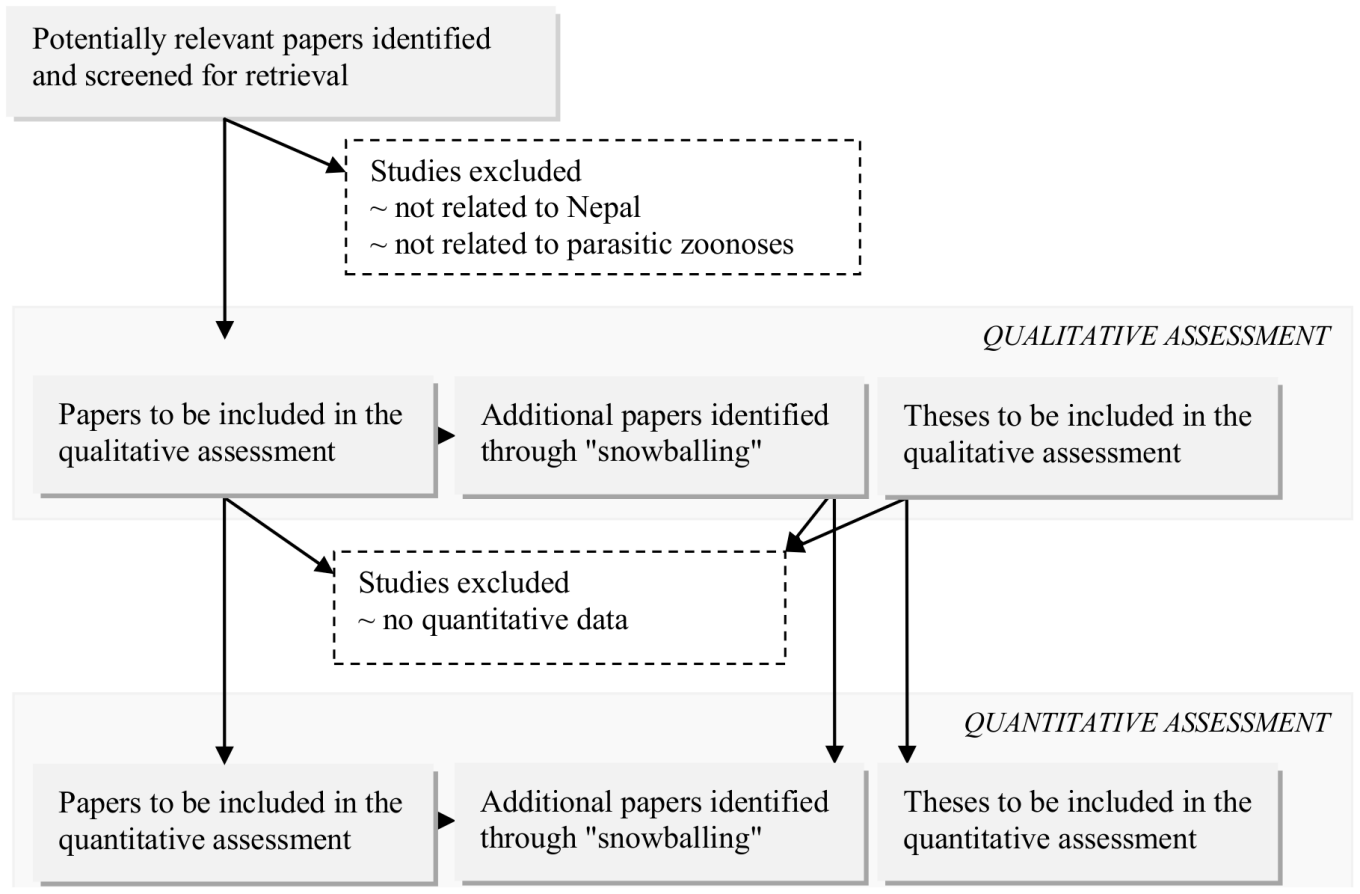
Generic flow diagram of applied search strategy.

In a second step, the retrieved titles were screened for eligibility by applying a set of predefined criteria to the titles and, if possible, to the abstracts and full texts. Only papers published in 1990 or later were considered eligible, and no restrictions were placed on the language of publication. For the qualitative assessment, documents were only excluded if they did not relate to the PZ in question, or if they did not pertain to Nepal or Nepalese patients. For the quantitative assessment, additional restrictions were put on the year of publication (between 2000 and 2012), the study setting and population (Nepalese patients infected in Nepal), and the type of information (quantitative, thus excluding case reports and case series). Finally, additional titles were sought for using forward and backward reference searches (so-called “snowballing”). In the forward reference search, the titles eligible for the qualitative assessment were entered in Google Scholar (http://scholar.google.com/) to obtain a list of articles citing the former. The latter were then screened using the same criteria as used in the initial searches. In the backward reference search, the reference lists of the initially retrieved eligible documents were hand-searched and the same criteria were applied. The forward and backward searches were repeated until no more new information could be retrieved. Figure 2 presents a generic flow diagram of this applied search strategy.

Relevant data on study setting, diagnostic methods and study results were extracted from all eligible articles, and entered in spread sheet documents for further use.

### Qualitative assessment

This initial assessment aimed at classifying the considered PZs according to their presumed endemicity status and data availability. To this end, we defined four categories:

- **Probably not endemic:** there is no direct or indirect local evidence and no direct evidence from neighboring countries;
- **Potentially endemic:** there is no direct or indirect local evidence, but there is direct evidence from neighboring countries; or, there is some direct local evidence, but of questionable nature, thus needing further confirmation;
- **Probably endemic & non-quantifiable:** there is direct or indirect local evidence; the burden cannot be quantified due to insufficient quantitative data or uncertainty in zoonotic potential or health effects;
- **Probably endemic & quantifiable:** there is direct or indirect local evidence; the burden can be quantified.

Additionally, information regarding the zoonotic nature of potentially zoonotic parasites was considered, with respect to alternative (dominant) anthroponotic transmission.

### Quantitative assessment

Where possible, the prevalence of each PZ classified as “probably endemic & quantifiable” was modeled using a random effects meta-analysis in a Bayesian framework. In this model, it is assumed that the number of positive samples *x_i_* in each study results from a binomial distribution with sample size *n_i_* and a study-specific true prevalence *θ_i_*, which is in its turn the result of an overall true prevalence *π* and a random study effect. The study effect is assumed to be normally distributed with mean zero and variance τ^2^. The prior distribution of τ^2^ is Gamma with scale and shape parameter equal to 1, while a Normal distribution with mean 0 and precision 0.001 was used as prior for the logit-transformed true prevalence. Markov chain Monte Carlo methods are used to fit the model. More information on the meta-analysis model is provided in Supporting Information S2.

If data allowed, the health impact of the concerning PZs was also quantified as the number of incident cases, deaths and DALYs. The DALY metric is a summary measure of public health, widely used in disease burden assessments and cost-effectiveness analyses [6], [7]. DALYs represent the overall number of healthy life years lost due to morbidity and mortality, hereby facilitating comparisons between diseases, and between countries and regions. The standard DALY formulas are:

DALY = YLD + YLL

YLD = Number of cases * Duration * Disability Weight

YLL = Number of deaths * Life expectancy at age of death

Calculation of DALYs was done using the standard formulas, and implemented in a fully stochastic framework using the DALY Calculator in R [15]. Supporting Information S3 presents the disease models and input distributions used for assessing the burden of the concerned PZs. We calculated undiscounted and unweighted DALYs, based on the Coale-Demeny model life table West, as our base case scenario. However, in order to enhance comparability of our estimates to estimates made by other authors, we performed scenario analyses by varying the time discount rate from 0% to 3%, by including age weighting, and by using the life expectancy table developed for the GBD 2010 study [7]. These different scenarios were denoted by DALY_{*K*,*r*}_, with *K* equal to 0 for unweighted DALYs and equal to 1 for age-weighted DALYs, and with *r* the time discount rate. For all scenarios, results were calculated at the population level (i.e., absolute number of DALYs per year) and at the individual level (i.e., relative number of DALYs per symptomatic case). Incident cases, deaths and DALYs were calculated for reference year 2006, i.e., the midpoint of the eligible publication period, 2000–2012. The total population size for 2006 was calculated as the mean of the population sizes estimated in the 2001 and 2011 censuses. The age and sex distribution of the 2006 population was derived from the 2006 Nepal Demographic and Health Survey [16]. Table 2 presents the resulting population sizes used in the calculations.

**Table 2:** 2006 age and sex specific population sizes used in the calculation of incident cases, deaths and DALYs.

	Male	Female	Total
**0–4**	1,766,025	1,673,583	**3,439,608**
**5–14**	3,655,548	3,556,364	**7,211,913**
**15–44**	4,668,234	6,331,723	**10,999,957**
**45–59**	1,259,682	1,464,385	**2,724,068**
**60+**	975,636	920,471	**1,896,107**
**All ages**	**12,325,126**	**13,946,527**	**26,271,653**

### Ethics statement

The data collection activities required for this study were approved by the ethical review board of the Nepal Health Research Council (Ramshahpath, Kathmandu, Nepal) and of the Ghent University Hospital (Ghent, Belgium; registration number B670201111932).

## Results

### Systematic review

For all twenty considered PZs, we identified 267 unique peer-reviewed documents and 50 unique dissertations. All identified documents were published in English. Table 3 summarizes the results of the systematic review for each considered PZ.

**Table 3:** Retrieved documents (total, retained).

Parasitic zoonosis	Total unique titles	Retained titles							
		*Qualitative assessment*				*Quantitative assessment*			
		Literature	Snowball	Thesis	Total	Literature	Snowball	Thesis	Total
Alveolar echinococcosis	3	1	0	0	**1**	0	0	0	**0**
Angiostrongylosis	3	0	0	0	**0**	0	0	0	**0**
*Anisakidae* infections	0	0	0	0	**0**	0	0	0	**0**
Capillariosis	7	2	0	4	**6**	0	0	0	**0**
Cystic echinococcosis	34	14	1	7	**22**	0	0	2	**2**
Cysticercosis	58	46	12	4	**62**	9	4	3	**16**
Diphyllobothriosis	1	0	0	3	**3**	0	0	0	**0**
Dirofilariosis	4	0	0	0	**0**	0	0	0	**0**
Foodborne trematodoses	22	2	4	5	**11**	0	0	0	**0**
Gnathostomosis	3	0	0	1	**1**	0	0	0	**0**
Sparganosis	3	1	0	0	**1**	0	0	0	**0**
Taeniosis	36	12	8	13	**33**	6	4	11	**21**
Toxocarosis	8	3	0	5	**8**	0	0	0	**0**
Toxoplasmosis	35	14	4	3	**21**	5	1	3	**9**
Trichinellosis	5	4	0	2	**6**	0	0	0	**0**
Zoonotic intestinal helminth infection	154	83	21	27	**131**	34	12	19	**65**
Zoonotic intestinal protozoal infection	114	62	24	23	**109**	36	12	16	**64**
Zoonotic leishmaniosis	242	17	1	1	**19**	0	0	0	**0**
Zoonotic schistosomosis	20	3	0	2	**5**	0	0	0	**0**
Zoonotic trypanosomosis	7	0	0	0	**0**	0	0	0	**0**

### Burden assessment

Table 4 presents the results of the qualitative classification of PZs. Out of the twenty considered PZs, only *Anisakidae* infection, zoonotic sleeping sickness (trypanosomosis) and zoonotic schistosomosis were classified as probably not endemic as no direct or indirect evidence was found. Seven PZs were classified as potentially endemic, i.e., alveolar echinococcosis, angiostrongylosis, capillariosis, dirofilariosis, gnathostomosis, sparganosis and cutaneous leishmaniosis. The ten remaining PZs were considered probably endemic, and the burden of three of these, neurocysticercosis, congenital (but not acquired) toxoplasmosis and cystic echinococcosis, could be fully quantified in terms of incident cases, deaths and DALYs.

**Table 4:** Results of the qualitative assessment (in alphabetical order).

Probably endemic & quantifiable	Probably endemic & non-quantifiable	Potentially endemic	Probably not endemic
Cystic echinococcosis	Diphyllobothriosis	Alveolar echinococcosis	*Anisakidae* infections
Cysticercosis	Foodborne trematodoses	Angiostrongylosis	Zoonotic schistosomosis
Toxoplasmosis	Taeniosis*	Capillariosis	Zoonotic trypanosomosis
	Toxocarosis	Dirofilariosis	
	Trichinellosis	Gnathostomosis	
	Zoonotic intestinal helminth infections*	Sparganosis	
	Zoonotic intestinal protozoal infections*	Zoonotic leishmaniosis	

* *For these parasitic zoonoses, prevalence estimates were available.*

#### Potentially endemic parasitic zoonoses

No local evidence could be found for alveolar echinococcosis, angiostrongylosis, capillariosis, dirofilariosis, gnathostomosis, sparganosis. However, recent case reports of these diseases in India indicate that these might be, or become, endemic in Nepal as well. Furthermore, some local evidence has been reported on zoonotic leishmaniosis, but this information remains unconfirmed. If any of these potentially endemic PZs are indeed endemic to Nepal, their burden is probably limited to a few sporadic cases.

So far, no cases of alveolar echinococcosis have been reported from Nepal, although a case of alveolar echinococcosis in a monk having traveled to Nepal, India, and Singapore has been reported [17]. However, given the considerable burden of alveolar echinococcosis in Tibetan communities [18], and the presence of putative cases from India [19], [20], [21], there are likely to be some cases in Nepal as well [22].

Human angiostrongylosis, capillariosis, dirofilariosis, gnathostomosis and sparganosis result from accidental infection with parasites that mainly have rodents, canines or felines as definitive hosts. The latter hosts are common in Nepal, and *Capillaria* eggs have already been identified in dog, cat and monkey stool samples and environmental samples [23], [24], [25], [26], [27], [28], but it is unclear whether these were *C. aerophila*, or the clinically more important *C. hepatica* and *C. philippinensis*, which cause hepatic and intestinal capillariosis, respectively. Furthermore, *Spirometra* has been identified in stray dog stool samples from Kathmandu Valley [27], and Gewali [24] apparently found *Gnathostoma* eggs in water samples from Kathmandu. Human cases of these five PZs have not yet been reported from Nepal, but sporadic cases have been reported from India. Cases of eosinophilic meningitis due to *Angiostrongylus cantonensis* have been reported mainly from the southern Indian states [29], [30]. Only few cases of human intestinal and hepatic capillariosis have been reported from India so far [31], [32]. Ocular and subcutaneous manifestations of human dirofilariosis due to *Dirofilaria immitis* and *Dirofilaria repens* have been reported from southern states of India, but there have also been cases from the northern state of Punjab [33], [34]. Barua et al. [35] reported a case of *Gnathostoma spinigerum* in a patient from the northeastern Indian state of Meghalaya, while Mukherjee et al. [36] present a case of cutaneous gnathostomosis in a female from the northeastern Indian state of Manipur. Some sparganosis case reports from India have been published, including cerebral [37], hepatic [38] and visceral manifestations, the latter in a patient from Uttar Pradesh [39].

It is widely recognized that Nepal is endemic for *Leishmania donovani*, the causative agent of anthroponotic visceral leishmaniosis (AVL), locally known as kala-azar [40], [41], [42]. Although some studies have hinted at a possible zoonotic transmission route of *L. donovani* [43], [44], [45], we considered kala-azar as a purely anthroponotic parasitic disease, and excluded it from the current study. In addition to AVL, however, several reports have presented cases of cutaneous leishmaniosis [46], [47], [48], [49], [50], [51]. Although most of these cases have been imported, mostly from the Middle East, one report mentions a case of cutaneous leishmaniosis caused by *Leishmania major* in a woman not known to have lived outside Nepal [52], [53], [54]. The presence of *Phlebotomus papatasi*, a possible vector of *L. major* and *L. infantum* [43], [55], [56], [57], [58], [59], further suggests that Nepal might be (or become) endemic for zoonotic leishmaniosis [60].

#### Probably endemic parasitic zoonoses

Trichinellosis has been confirmed in pigs, but never in humans in Nepal. Serological and/or coprological evidence of human infections with *Toxocara*, *Diphyllobothrium*, foodborne trematodes (FBT), and *Taenia* exists, but the population impact of these PZs is probably too low to quantify, although certain groups might be at high risk. Although patent infections with intestinal helminths and protozoa are still very common, the health impact of *zoonotic* intestinal helminths and protozoa could not be assessed, due to uncertainty of zoonotic potential and health effects. On the other hand, the health impact of cysticercosis, toxoplasmosis and cystic echinococcosis was deemed quantifiable.

*Trichinella* infection has been serologically confirmed in pigs from Kathmandu [61], [62], although Karn et al. [63] could not find seropositives in a sample of 344 pigs slaughtered in five districts of the Central Development Region of Nepal (including Kathmandu). Larvae have so far not yet been found on digestion. No human cases have been reported from Nepal, although Joshi et al. [61] mention the undocumented occurrence of sporadic cases of human trichinellosis reported from medical hospitals, and a trichinellosis outbreak has been documented from the north Indian state of Uttarakhand [64].

In a serological ELISA study, a high proportion of Nepalese people (∼80%) appeared positive for *Toxocara* infection [65]. Recently, two children with eosinophilia were serodiagnosed with toxocarosis [66]. Furthermore, *Toxocara* spp. have been identified from dogs from Kathmandu [27], [67], [68], cats from Nawalparasi and Chitwan [26], and water samples from Kathmandu [24], [25].

*Diphyllobothrium* has been found in dog stool samples from Kathmandu [67] and in the intestine of common carp fingerlings from a fish farm near Kathmandu [69]. Thapa [70] reports finding *Diphyllobothrium* eggs in the stools of 18/62 (29.0%) and 2/90 (2.2%) people of the Bote and Darai ethnic communities, respectively. Both are marginalized communities from Chitwan, and mainly depend on agriculture and fishing.

Some Nepali studies have reported trematode eggs in human and animal stools. Eggs of *Fasciola* spp. have been reported from buffaloes in a number of studies [71], [72], [73], [74], [75]. In community-based studies conducted in Kavre and Chitwan, *Fasciola* spp. eggs were reported in human stools [76], [77]. In a study of diarrheal samples from Kathmandu, eggs resembling those of *Clonorchis sinensis* or *Opisthorchis* spp. were found [78], [79]. However, as identification was based on visual identification only, confirmation is not certain. Three out of 84 children with eosinophilia presenting at a university hospital in Kavre were serologically positive for fasciolosis [66], while in another case series on eosinophilia in children, paragonimosis was suggested as a possible cause, given that a significant proportion of patients had the habit of eating undercooked fresh water crab meat [80]. In India, human cases of *Fasciolopsis buski* have been described from the Nepal bordering states of Bihar [81], [82] and Uttar Pradesh [83].

Taeniosis, due to *Taenia solium*, *Taenia saginata* or *Taenia asiatica*, is commonly reported in Nepal. Different studies indicate low taeniosis prevalences in the general public and in clinical samples (<2%), although some papers hint at high prevalences in certain ethnic groups (10–50%) (Table S3-1 in Supporting Information S3). Higher taeniosis prevalence in certain groups is possible, as discussed by Prasad et al. [84] and Devleesschauwer et al. [85], although estimates of up to 50% are somewhat doubtful. Molecular studies have identified *T. asiatica* and *T. saginata* as causes of taeniosis [85], [86], although it is to be expected that *T. solium* also causes taeniosis in Nepal, given its presence in animal intermediate hosts [87]. Apart from rare complications such as gastrointestinal obstruction or inflammation, the health impact of taeniosis is minimal. So far, there has only been one report describing such complications in a Nepalese patient [88]. As neither the national nor the international literature give a clear view of the probability of developing such complications, it was decided that the health impact could not be quantified.

A large number of studies have assessed the prevalence of intestinal helminths and protozoa in Nepal (Table S3-2 and S3-3 in Supporting Information S3). Community-based studies mainly targeted school children, while hospital-based studies were mostly set in large urban referral hospitals. Fewer studies looked at intestinal helminthic and protozoal infestations in HIV-AIDS patients. However, a meaningful quantification of the public health impact of zoonotic intestinal helminths and protozoa was deemed impossible, due to the uncertainty regarding the extent to which these infections are truly zoonotic and the uncertainty regarding the health effects of zoonotic species [1]. Indeed, the limited available data suggest that intestinal helminth infections are mainly due to anthroponotic species. In the large study on genetic influences of helminth susceptibility in the Jirel population of Jiri, Dolakha, only *Ascaris lumbricoides* was reported [89], [90]. Fecal cultures to identify hookworm larvae so far only revealed the anthroponotic hookworm species *Ancylostoma duodenale* and *Necator americanus* [91], [92]. The zoonotic relevance of intestinal protozoa remains less clear, even though genetic characterization of *Giardia* and *Cryptosporidium* from Nepal has been performed [93], [94], [95], [96]. The zoonotic potential of *Blastocystis* appears to be best studied [97], [98], [99], yet there is large uncertainty about the prevalence of human infection given the limited number of studies, as well as substantial doubt regarding its pathogenic nature [100].

Human cysticercosis in Nepalese people has been described since the early 1990s, mainly through reports of patients with neurocysticercosis (NCC) [101], [102], [103], [104], [105], [106], [107], [108], [109], [110], [111], ocular cysticercosis [112], [113], [114], [115], [116] and muscular and soft tissue cysticercosis [117], [118], [119], [120], [121], [122], [123], [124], [125]. Its public health impact however did not receive full attention until the 2000s. Hospital-based studies indicate NCC prevalences in seizure patients ranging from 7% to 73% (Table S3-5 in Supporting Information S3). The majority of these studies applied neuro-imaging. Two studies report the prevalence of NCC in hydrocephalus patients, indicating a prevalence of 1–2% [126], [127], while a recent study indicates NCC prevalence of ∼5% in patients with chronic headache [128]. A case series of three Nepalese intraventricular NCC patients molecularly identified the removed lesions as *T. solium* [129].

The majority of population-based studies on *Toxoplasma gondii* seroprevalence are from the 1990s [130], [131], [132], [133], [134], apart from two recent studies [135], [136]. *T. gondii* seroprevalence has also been studied in women with bad obstetric history [137], [138], [139], [140], patients with HIV/AIDS [139], [141], [142], [143], [144], ocular disorders [139] and hydrocephalus [126]. Apart from a recent case description [145], however, there appears to be no direct evidence on the impact of congenital toxoplasmosis, which is likely to represent the highest population burden [1]. As a result, it is only possible to obtain an indirect view of the impact of congenital toxoplasmosis in Nepal through population-based seroprevalence data.

Cystic echinococcosis has traditionally mostly been studied in livestock [27], [146], [147], [148], [149], [150], [151]. The few data in dogs indicate higher prevalence in areas where livestock is slaughtered [149], [152]. Since the 2000s, various case reports have been published on human hydatidosis [153], [154], [155], [156], [157], [158], [159], [160], [161]. Hospital register studies for CE cases have found low incidences [147], [162], [163]. So far, genotyping studies have revealed the presence of G1, the sheep strain in humans [164], dogs and livestock [149], [165], [166], G5 (cattle strain) in livestock [165] and G6 (camel strain) in humans [165].

**Figure 3:**
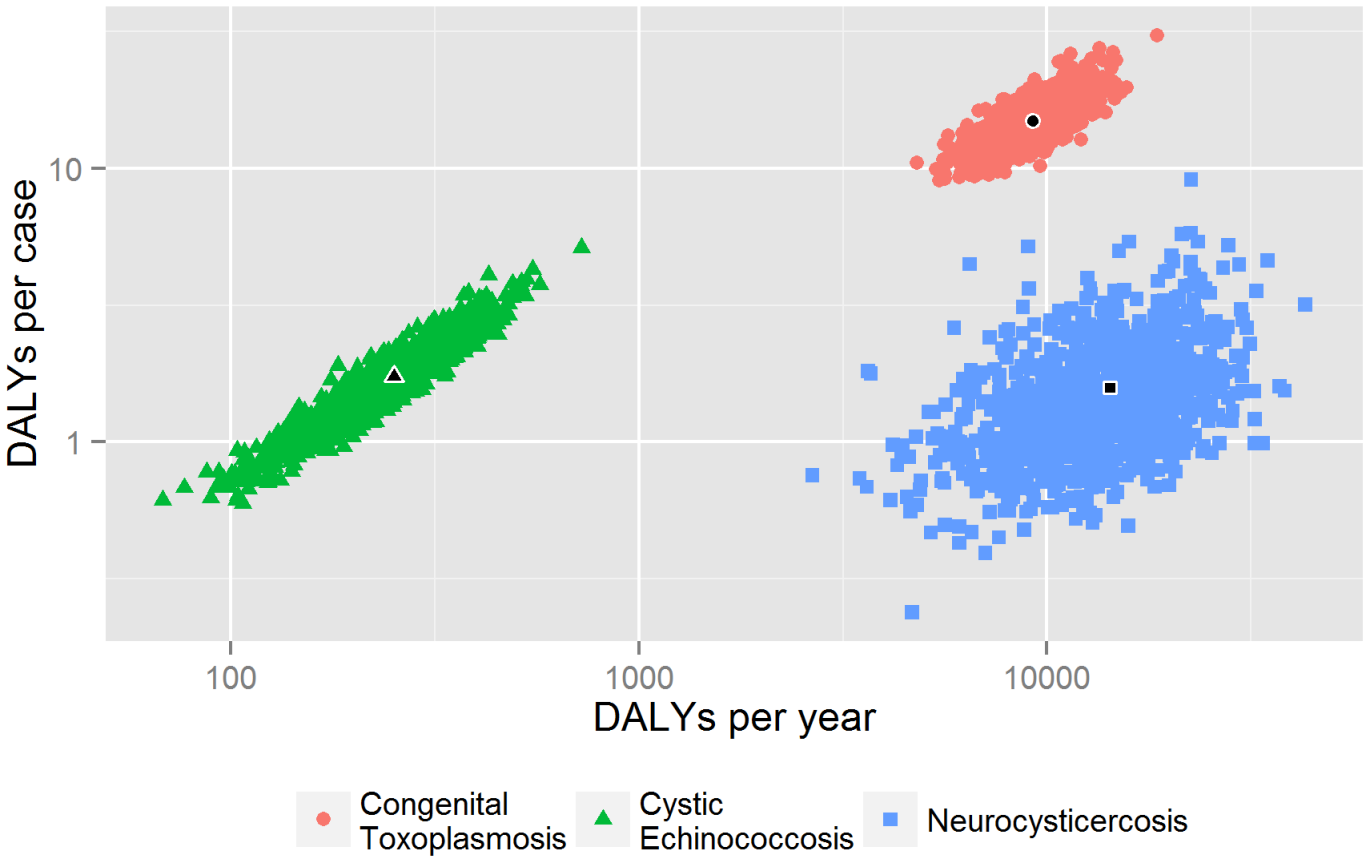
Population-level (DALYs_{0,0}_ per year) versus individual-level burden (DALYs_{0,0}_ per symptomatic case) in Nepal, 2006; the scatterplots represent 1000 random samples from each distribution, with the black symbol representing the centroid; both axes are on a log10 scale.

Quantitative assessment. For intestinal infestations with *Taenia* spp., helminths and protozoa, we were able to estimate prevalence based on a random effects meta-analysis (Table 5). For neurocysticercosis, congenital toxoplasmosis and cystic echinococcosis we could estimate the number of incident cases, deaths and DALYs (Table 6; see Supporting Information S3 for more details on underlying data driving these estimates). Figure 3 visualizes the estimated burden at population and individual level, taking into account the uncertainty resulting from the parameter uncertainties, as suggested by Havelaar et al. [167]. Congenital toxoplasmosis has both a high population and patient burden, while neurocysticercosis is relatively less important at the patient level, but equally important at the population level. Cystic echinococcosis appears less important at both levels.

**Table 5:** Quantitative assessment – occurrence of intestinal parasites.

Intestinal parasite	Number of datasets	Estimated prevalence (%)		
		Mean	95% Range*	Distribution
***Community-based studies***				
*Taenia* spp.	15	3.4	0.7–8.1	Beta(2.977, 84.058)
*Ascaris* spp.	37	15.6	10.6–21.4	Beta(26.936, 145.189)
*Trichuris* spp.	36	11.2	6.4–17.1	Beta(14.545, 115.807)
Hookworm	35	10.4	5.9–15.9	Beta(14.476, 125.337)
*Giardia* spp.	28	8.9	6.2–12.0	Beta(32.089, 330.444)
*Cryptosporidium* spp.	12	0.6	0.2–1.4	Beta(4.165, 641.569)
*Blastocystis hominis*	5	6.9	1.5–15.7	Beta(3.137, 42.457)
***Hospital-based studies***				
*Taenia* spp.	8	0.5	0.1–1.0	Beta(4.575, 977.996)
*Ascaris* spp.	25	3.4	1.8–5.5	Beta(12.621, 353.634)
*Trichuris* spp.	25	1.0	0.4–2.0	Beta(5.588, 550.789)
Hookworm	25	1.5	0.7–2.7	Beta(8.954, 577.751)
*Giardia* spp.	28	5.5	3.8–7.6	Beta(29.778, 507.152)
*Cryptosporidium* spp.	17	1.7	0.6–3.3	Beta(6.077, 352.688)
*Blastocystis hominis*	7	1.2	0.0–4.5	Beta(0.948, 77.444)
***HIV-aids patients***				
*Ascaris* spp.	4	2.1	0.0–9.2	Beta(0.671, 30.91)
*Trichuris* spp.	4	4.3	0.1–15.5	Beta(0.935, 21.065)
Hookworm	4	3.1	0.0–12.3	Beta(0.79, 24.688)
*Giardia* spp.	5	5.6	1.7–11.6	Beta(4.32, 73.437)
*Cryptosporidium* spp.	8	6.4	2.7–11.7	Beta(7.069, 102.905)
*Blastocystis hominis*	3	2.8	0.1–9.4	Beta(1.157, 40.646)

* *Defined as the 2.5^th^ and 97.5^th^ percentile of the concerned distribution.*

**Table 6:** Quantitative assessment – disease impact*.

Parasitic zoonosis	Neurocysticercosis^a^	Congenital toxoplasmosis^b^	Cystic echinococcosis^c^	Total
**Incident cases [95%CrI** ** **]**	—	1396 [1058–1780]	145 [114–179]	—
**Incident symptomatic cases** *** **[95%CrI** ** **]**	10,618 [3304–22,296]	626 [473–813]	145 [114–179]	11,389 [4083–23,045]
**Deaths [95%CrI** ** **]**	163 [39–378]	60 [27–105]	3 [0–7]	225 [93–442]
**DALY_{0,0}_ [95%CrI** ** **]**	14,268 [5450–27,694]	9255 [6135–13,292]	251 [105–458]	23,773 [14,094–37,719]
**DALY_{0,0}_/1000 [95%CrI** ** **]**	0.543 [0.207–1.054]	0.352 [0.234–0.506]	0.010 [0.004–0.017]	0.905 [0.536–1.436]
**DALY_{0,0}_/symptomatic case [95%CrI** ** **]**	1.581 [0.576–4.047]	14.934 [10.128–21.796]	1.741 [0.737–3.243]	—
**DALY_{1,0.03}_ [95%CrI** ** **]**	10,924 [4270–21,301]	3964 [2648–5653]	204 [116–323]	15,092 [8215–25,546]
**DALY_{0,0.03}_ [95%CrI** ** **]**	8916 [3569–17043]	3553 [2359–5098]	174 [96–277]	12,642 [7046–20,791]
**DALY_{0,0}–GBD2010 Life Expectancy_ [95%CrI** ** **]**	14,994 [5668–29,273]	9673 [6347–14,017]	263 [106–486]	24,930 [14,706–39,702]

* *Scenarios are denoted as DALY_{age weighting constant, discount rate}_.*

** *Credibility Interval.*

*** *Incident symptomatic cases are the sum of all clinical manifestations across all incident cases.*

a *The clinical manifestations incorporated in the neurocysticercosis DALY estimates were epilepsy and death; note that the number of incident neurocysticercosis cases was not calculated, as the estimation started from the incidence of epilepsy (see Supporting Information S3).*

b *The clinical manifestations incorporated in the congenital toxoplasmosis DALY estimates were chorioretinitis at birth, chorioretinitis later in life, hydrocephalus, intracranial calcifications, central nervous system abnormalities, fetal death and neonatal death.*

c *The clinical manifestations incorporated in the cystic echinococcosis DALY estimates were post-surgical recovery (with rehabilitation and possible worrying), substantial post-surgical conditions, post-surgical recurrent disease, post-surgical death, and an average health state for non-reported cases; no burden was attributed to healthcare seeking cases that were not treated surgically.*

## Discussion

As disease burden estimates are of increasing importance for policy making and evaluation, the need for such estimates becomes eminent. In the late 1990s, the World Bank commissioned a comprehensive analysis of health care delivery in Nepal. Several recommendations were made for the further development of the Nepalese health sector, one of which was the establishment of priorities [168]. These recommendations were carried forward in the development of the *Nepal Health Sector Programmes* (NHSP), short-term strategic frameworks for the further development of the health sector. Since then, disease burden is recognized as one of the bases for setting program priorities [5]. However, when routine surveillance systems are performing poorly and baseline epidemiological studies are rare, these estimates are not readily available [169]. In this paper, we present the first comprehensive systematic review of the burden of PZs in Nepal. Information was sought from the international and national peer-reviewed scientific literature, and an important source of information was found in dissertations. The information found allowed qualitative assessment of the twenty PZs considered. However, quantitative estimates of prevalence or disease burden were possible for only a few.

Nepal is considered endemic for at least ten PZs, and might be endemic for seven others. Most of these diseases probably only have a small public health impact. However, neurocysticercosis and congenital toxoplasmosis are likely to impose an important burden to public health. Indeed, if we compare with the three “major” infectious diseases, we see that the estimated burden due to major clinical manifestations of three PZs, with in total 0.57 DALY_{1,0.03}_ per 1000 people, is higher than that of the WHO 2004 GBD estimate for malaria (0.05 DALY_{1,0.03}_ per 1000), comparable to that for HIV/AIDS (0.74 DALY_{1,0.03}_ per 1000), but substantially lower than that for tuberculosis (5.45 DALY_{1,0.03}_ per 1000) [6]. These comparisons suggest that greater attention for PZs in Nepal is warranted. Toxoplasmosis is for instance not reported in any official Nepalese data collection system, and cysticercosis and toxoplasmosis were not considered in the WHO 2004 GBD update [6]. As a result, the incidence of congenital toxoplasmosis remains a critical data gap, and considerable uncertainties remain regarding the epilepsy prevalence and proportion of neurocysticercosis-associated epilepsy. Data on the zoonotic potential of intestinal helminths and protozoa and their health effects are lacking, although these infections may represent a considerable additional health burden.

In our study, certain methodological choices were made with as a consequence certain limitations. First, instead of applying strict inclusion/exclusion criteria, we aimed at collecting as much relevant information as possible. Inherently, this leads to large heterogeneity in the collected quantitative data. As a result, our burden estimates have large uncertainty intervals, making it for instance impossible to statistically distinguish the burden of neurocysticercosis and congenital toxoplasmosis. For congenital toxoplasmosis, as no direct evidence was available, we estimated the incidence based on a single age-specific seroprevalence study. Clearly, this puts an important constraint on the representativeness of our resulting burden estimate. Direct evidence on the incidence of congenital toxoplasmosis (e.g., through serological studies on newborns), preferably obtained through a multi-center study, is therefore needed to confirm our burden estimate.

Second, uncertainty was introduced by the selection and valuation of the clinical outcomes for the three diseases. We based our disease models on published studies [170]–[173], but note that other authors applied alternative ones. For instance, Bhattarai et al. [174] also included severe headaches in their assessment of the burden of neurocysticercosis in Mexico, whereas this was deemed infeasible in our study. Likewise, the disability weights assigned to the different included clinical outcomes were derived from earlier studies [170]–[173], in order to enhance comparability with those studies. Nevertheless, other studies, including the GBD studies, are less transparent about their applied disease models and disability weights, impeding unambiguous comparisons. For instance, the GBD 2010 study estimated the number of DALY_{0,0}_ in Nepal to be 667 [141–2073] for “echinococcosis” and 4220 [2786–6022] for cysticercosis [175]. Given a lack of knowledge on the disease models, disability weights and data behind these estimates, it is difficult to assess the reason for any differences or similarities with our estimates. Toxoplasmosis and other PZs appear to be absent from the GBD 2010 study.

Third, subjective methodological choices regarding the calculation of DALYs may lead to further uncertainty. We tried to deal with this source of uncertainty by calculating DALYs under different common sets of normative assumptions, i.e., no discounting and age weighting, 3% time discounting and no age weighting, 3% time discounting and age weighting; and by calculating DALYs based on both the Coale-Demeny model life table West and the new GBD 2010 life expectancy table. As expected, time discounting led to smaller burden estimates. The difference between both life tables was minimal.

In addition, this study focused on the population burden of PZs. Some PZs, however, might have an important individual burden, even though their population burden is negligible or small. Likewise, the burden suffered by specific sub-populations (e.g., caste or ethnic groups), might be much higher than average population burden [85].

Finally, due to a lack of time and resources, we had to place restrictions on the nature of the diseases to be studied, and on the nature of the burden estimates to be generated. Indeed, this study only focuses on the burden of *parasitic* zoonoses. However, as means for interventions are poor, future integrated control should be packaged by, for instance, simultaneously controlling cystic echinococcosis, brucellosis and rabies. This analysis should therefore be extended to the burden of bacterial and viral zoonoses in Nepal. By quantifying the burden in terms of incidence, mortality and DALYs, we also focused on the *health* impact of the concerned diseases. Some PZs might have an important economic impact, for example in terms of livestock health, or might reduce psycho-social wellbeing in a way not captured by the applied metrics. Truly evidence-informed priority setting and decision making should take in account all these aspects of disease burden, implying that our estimates should be complemented by others.

Despite these limitations, this study has identified the most important PZs for Nepal, as far as existing data allows. The quantitative estimates of disease burden for three of these diseases suggest that PZs deserve greater attention and more intensive surveillance. As population and disease transmission dynamics change over time, disease burden changes dynamically as well. Therefore, the presented results should be updated regularly, and this exercise should be extended to other groups of neglected diseases or even to a full national burden of disease study. We therefore hope that this study will stimulate further research, so that the overall human health burden in Nepal can be better characterized. In the long term, however, continued efforts to improve surveillance and database system at the local level should enable truly monitoring of disease burden over time.

## Supporting Information

Checklist S1 PRISMA checklist.
(DOC)

Supporting Information S1 Search strategy.
(DOC)

Supporting Information S2 Bayesian random-effects meta-analysis.
(DOC)

Supporting Information S3 Quantitative burden assessment.
(DOCX)

## References

[1] Torgerson PR, Macpherson CN (2011) The socioeconomic burden of parasitic zoonoses: global trends. Vet Parasitol 182: 79–95. 21862222 10.1016/j.vetpar.2011.07.017

[2] Hotez PJ, Alibek K (2011) Central Asia's hidden burden of neglected tropical diseases. PLoS Negl Trop Dis 5: e1224. 21980541 10.1371/journal.pntd.0001224 PMC3181239

[3] Torgerson PR (2013) One world health: Socioeconomic burden and parasitic disease control priorities. Vet Parasitol 10.1016/j.vetpar.2013.04.004 23628712

[4] Murrell KD (1991) Economic losses resulting from food-borne parasitic zoonoses. Southeast Asian J Trop Med Public Health 22 Suppl: 377–381. 1822931

[5] Ministry of Health and Population (2010) Nepal Health Sector Programme – Implementation Plan II (NHSP-IP 2) 2010–2015. Kathmandu, Nepal. Available: http://www.unfpa.org/sowmy/resources/docs/library/R090_MOHNepal_2010_NHSP-IP-II_Final_Apr2010.pdf. Accessed 12 July 2013.

[6] Mathers C, Fat DM, Boerma J (2008) The global burden of disease: 2004 update: World Health Organization.

[7] Murray CJ, Vos T, Lozano R, Naghavi M, Flaxman AD, et al (2012) Disability-adjusted life years (DALYs) for 291 diseases and injuries in 21 regions, 1990–2010: a systematic analysis for the Global Burden of Disease Study 2010. Lancet 380: 2197–2223. 23245608 10.1016/S0140-6736(12)61689-4

[8] Vanderelst D, Speybroeck N (2010) Quantifying the lack of scientific interest in neglected tropical diseases. PLoS Negl Trop Dis 4: e576. 20126268 10.1371/journal.pntd.0000576 PMC2811171

[9] Trägård A, Shrestha IB (2010) System-wide effects of Global Fund investments in Nepal. Health Policy and Planning 25: i58–i62. 20966112 10.1093/heapol/czq061

[10] Nepal Health Sector Support Programme & Ministry of Health and Population (2011) Consensus building workshop on strengthening Health Management Information System (HMIS): Report outlining workshop findings, recommendations for Government & NHSSP support. Kathmandu, Nepal. 30 p.

[11] Thapa A (2007) Current situation in mortality statistics in Nepal. Kathmandu, Nepal: Ministry of Health and Population. 135 p.

[12] Dorny P, Praet N, Deckers N, Gabriel S (2009) Emerging food-borne parasites. Vet Parasitol 163: 196–206. 19559535 10.1016/j.vetpar.2009.05.026

[13] World Health Organization (2006) The control of neglected zoonotic diseases: A route to poverty alleviation. Available: http://www.who.int/zoonoses/Report_Sept06.pdf. Accessed 12 July 2013.

[14] World Health Organization (2010) First WHO report on neglected tropical diseases: Working to overcome the global impact of neglected tropical diseases. Available: http://whqlibdoc.who.int/publications/2010/9789241564090_eng.pdf. Accessed 12 July 2013.

[15] Devleesschauwer B, McDonald S, Haagsma J, Praet N, Havelaar A, Speybroeck N (2013) DALY: The DALY Calculator - A GUI for stochastic DALY calculation in R. R package version 1.2.0. Available: http://cran.r-project.org/package=DALY. Accessed 12 July 2013.

[16] Ministry of Health and Population (MOHP) [Nepal], New ERA, Macro International Inc (2007) Nepal Demographic and Health Survey 2006. Kathmandu: Ministry of Health and Population, New ERA, and Macro International Inc. 291 p. Available: http://www.measuredhs.com/pubs/pdf/FR191/FR191.pdf. Accessed 12 July 2013.

[17] Huang J, Wu YM, Liang PC, Lee PH (2004) Alveolar hydatid disease causing total occlusion of the inferior vena cava. J Formos Med Assoc 103: 633–636. 15340664

[18] Craig PS, Li T, Qiu J, Zhen R, Wang Q, et al (2008) Echinococcosis and Tibetan communities. Emerg Infect Dis 14: 1674–1675. 18826849 10.3201/eid1410.071636 PMC2609884

[19] Taneja K, Gothi R, Kumar K, Jain S, Mani RK (1990) Peritoneal *Echinococcus multilocularis* infection: CT appearance. J Comput Assist Tomogr 14: 493–494. 2335629

[20] Shaw AK, Gambhir RP, Chaudhry R, Jaiswal SS (2010) *Ecchinococcus multilocularis* causing alveolar hydatid disease liver: a rare occurrence in Indian subcontinent. Trop Gastroenterol 31: 119–120. 20862989

[21] Tyagi DK, Balasubramaniam S, Sawant HV (2010) Primary calcified hydatid cyst of the brain. J Neurosci Rural Pract 1: 115–117. 21808518 10.4103/0976-3147.71729 PMC3139339

[22] Torgerson PR, Keller K, Magnotta M, Ragland N (2010) The global burden of alveolar echinococcosis. PLoS Negl Trop Dis 4: e722. 20582310 10.1371/journal.pntd.0000722 PMC2889826

[23] Rai SK, Uga S, Ono K, Rai G, Matsumura T (2000) Contamination of soil with helminth parasite eggs in Nepal. Southeast Asian J Trop Med Public Health 31: 388–393. 11127345

[24] Gewali L (2002) Bacteriological and helminthological assessment of the drinking water quality of Ward-19, KMC [MSc dissertation]. Kathmandu: Tribhuvan University. 80 p.

[25] Thapa S (2002) Assessment of biological contamination and waterborne helminthic parasites in drinking water sources of Ward no 20, KMC [MSc dissertation]. Kathmandu: Tribhuvan University. 72 p.

[26] Khanal BP (2004) Studies on some intestinal helminth parasites of *Felis catus* (Linnaeus, 1758) from Nawalparasi and Chitwan districts of Nepal [MSc dissertation]. Kathmandu: Tribhuvan University. 59 p.

[27] Manandhar S, Horchner F, Morakote N, Kyule MN, Baumann MP (2006) Occurrence of hydatidosis in slaughter buffaloes (*Bos bubalis*) and helminths in stray dogs in Kathmandu Valley, Nepal. Berl Munch Tierarztl Wochenschr 119: 308–311. 17009714

[28] Dhoubhadel M (2007) Prevalence of intestinal helminth parasites in Rhesus monkey (*Macaca mulatta*) of Swoyambhu and Nilbarahi area of Kathmandu Valley [MSc dissertation]. Kathmandu: Tribhuvan University. 58 p.

[29] Malhotra S, Mehta DK, Arora R, Chauhan D, Ray S, et al (2006) Ocular angiostrongyliasis in a child–first case report from India. J Trop Pediatr 52: 223–225. 16186138 10.1093/tropej/fmi092

[30] Panackel C, Cherian G, Vijayakumar K, Sharma RN (2006) Eosinophilic meningitis due to *Angiostrongylus cantonensis* . Indian J Med Microbiol 24: 220–221. 16912445

[31] Vasantha PL, Girish N, Leela KS (2012) Human intestinal capillariasis: a rare case report from non-endemic area (Andhra Pradesh, India). Indian J Med Microbiol 30: 236–239. 22664447 10.4103/0255-0857.96708

[32] Nabi F, Palaha HK, Sekhsaria D, Chiatale A (2007) *Capillaria hepatica* infestation. Indian Pediatr 44: 781–782. 17998580

[33] Gautam V, Rustagi IM, Singh S, Arora DR (2002) Subconjunctival infection with *Dirofilaria repens* . Jpn J Infect Dis 55: 47–48. 12082307

[34] Chopra R, Bhatti SM, Mohan S, Taneja N (2012) *Dirofilaria* in the anterior chamber: a rare occurrence. Middle East Afr J Ophthalmol 19: 349–351. 22837635 10.4103/0974-9233.97965 PMC3401811

[35] Barua P, Hazarika NK, Barua N, Barua CK, Choudhury B (2007) Gnathostomiasis of the anterior chamber. Indian J Med Microbiol 25: 276–278. 17901651 10.4103/0255-0857.34775

[36] Mukherjee A, Ahmed NH, Samantaray JC, Mirdha BR (2012) A rare case of cutaneous larva migrans due to *Gnathostoma* sp. Indian J Med Microbiol 30: 356–358. 22885209 10.4103/0255-0857.99505

[37] Rengarajan S, Nanjegowda N, Bhat D, Mahadevan A, Sampath S, et al (2008) Cerebral sparganosis: a diagnostic challenge. Br J Neurosurg 22: 784–786. 18661311 10.1080/02688690802088073

[38] Khurana S, Appannanavar S, Bhatti HS, Verma S (2012) Sparganosis of liver: a rare entity and review of literature. BMJ Case Rep 2012. 10.1136/bcr-2012-006790 PMC4543914 23220827

[39] Duggal S, Mahajan RK, Duggal N, Hans C (2011) Case of sparganosis: a diagnostic dilemma. Indian J Med Microbiol 29: 183–186. 21654118 10.4103/0255-0857.81789

[40] Stauch A, Sarkar RR, Picado A, Ostyn B, Sundar S, et al (2011) Visceral leishmaniasis in the Indian subcontinent: modelling epidemiology and control. PLoS Negl Trop Dis 5: e1405. 22140589 10.1371/journal.pntd.0001405 PMC3226461

[41] Pun SB, Pandey K, Shah R (2013) A series of case reports of autochthonous visceral leishmaniasis, mostly in non-endemic hilly areas of Nepal. Am J Trop Med Hyg 88: 227–229. 23249686 10.4269/ajtmh.2012.12-0502 PMC3583309

[42] Uranw S, Meheus F, Baltussen R, Rijal S, Boelaert M (2013) The household costs of visceral leishmaniasis care in south-eastern Nepal. PLoS Negl Trop Dis 7: e2062. 23469298 10.1371/journal.pntd.0002062 PMC3585119

[43] Burniston I, Roy L, Picado A, Das M, Rijal S, et al (2010) Development of an enzyme-linked immunosorbent assay to identify host-feeding preferences of *Phlebotomus* species (Diptera: Psychodidae) in endemic foci of visceral leishmaniasis in Nepal. J Med Entomol 47: 902–906. 20939388 10.1603/me09184

[44] Khanal B, Picado A, Bhattarai NR, Van Der Auwera G, Das ML, et al (2010) Spatial analysis of *Leishmania donovani* exposure in humans and domestic animals in a recent kala azar focus in Nepal. Parasitology 137: 1597–1603. 20459877 10.1017/S0031182010000521

[45] Bhattarai NR, Van der Auwera G, Rijal S, Picado A, Speybroeck N, et al (2010) Domestic animals and epidemiology of visceral leishmaniasis, Nepal. Emerg Infect Dis 16: 231–237. 20113552 10.3201/eid1602.090623 PMC2958000

[46] Karki P, Parija SC, George S, Das ML, Koirala S (1997) Visceral leishmaniasis with cutaneous ulcer or cutaneous leishmaniasis in Nepal. Southeast Asian J Trop Med Public Health 28: 836–837. 9656411

[47] Parija SC, Jacob M, Karki BM, Sethi M, Karki P, et al (1998) Cutaneous leishmaniasis in Nepal. Southeast Asian J Trop Med Public Health 29: 131–132. 9740286

[48] Joshi A, Agrawal S, Garg VK, Thakur A, Agarwalla A, et al (2000) Severe mucosal involvement in a patient with cutaneous leishmaniasis from Nepal. Int J Dermatol 39: 317–318. 10809989 10.1046/j.1365-4362.2000.00930-4.x

[49] Pandey BD, Babu E, Thapa S, Thapa LB (2006) First case of cutanous leishmaniasis in Nepalese patient. Nepal Medical College Journal: NMCJ 8: 213. 17203834

[50] Neupane S, Sharma P, Kumar A, Paudel U, Pokhrel DB (2008) Cutaneous leishmaniasis: report of rare cases in Nepal. Nepal Med Coll J 10: 64–67. 18700634

[51] Kayastha B, Shrestha P, Shrestha R, Jahan R (2009) Cutaneous leishmaniasis: a case report. Nepal Journal of Dermatology, Venereology & Leprology 8: 27–30.

[52] Kumar R, Ansari NA, Avninder S, Ramesh V, Salotra P (2008) Cutaneous leishmaniasis in Nepal: *Leishmania major* as a cause. Trans R Soc Trop Med Hyg 102: 202–203. 18177679 10.1016/j.trstmh.2007.10.017

[53] Kalra NL, Arya SC (2008) Comment on: Cutaneous leishmaniasis in Nepal: *Leishmania major* as a cause. Trans R Soc Trop Med Hyg 102: 618 author reply 618–619. 18353409 10.1016/j.trstmh.2008.02.003

[54] Kumar R, Ansari NA, Avninder S, Ramesh V, Salotra P (2008) Reply to comment on: Cutaneous leishmaniasis in Nepal: *Leishmania major* as a cause. Trans R Soc Trop Med Hyg 102: 618–619. 18177679 10.1016/j.trstmh.2007.10.017

[55] Joshi A, Banjara M, Pokhrel S, Jimba M, Singhasivanon P, et al (2006) Elimination of visceral leishmaniasis in Nepal: pipe-dreams and possibilities. Kathmandu Univ Med J (KUMJ) 4: 488–96. 18603960

[56] Pandey K, Pant S, Kanbara H, Shuaibu MN, Mallik AK, et al (2008) Molecular detection of Leishmania parasites from whole bodies of sandflies collected in Nepal. Parasitol Res 103: 293–297. 18415124 10.1007/s00436-008-0967-7

[57] Hamarsheh O, Presber W, Yaghoobi-Ershadi MR, Amro A, Al-Jawabreh A, et al (2009) Population structure and geographical subdivision of the *Leishmania major* vector *Phlebotomus papatasi* as revealed by microsatellite variation. Med Vet Entomol 23: 69–77. 19239616 10.1111/j.1365-2915.2008.00784.x

[58] Gidwani K, Picado A, Rijal S, Singh SP, Roy L, et al (2011) Serological markers of sand fly exposure to evaluate insecticidal nets against visceral leishmaniasis in India and Nepal: a cluster-randomized trial. PLoS Negl Trop Dis 5: e1296. 21931871 10.1371/journal.pntd.0001296 PMC3172194

[59] Bhandari G, Angdembe M, Rijal S, Boelaert M (2011) Will visceral leishmaniasis be eliminated from Nepal? A review of recent (1994–2006) control efforts. Nepal Medical College journal: NMCJ 13: 220. 22808821

[60] Schwarz D, Andrews J, Gauchan B (2011) Visceral leishmaniasis in far western Nepal: another case and concerns about a new area of endemicity. Am J Trop Med Hyg 84: 508. 21363996 10.4269/ajtmh.2011.11-0021 PMC3042834

[61] Joshi DD, Moller LN, Maharjan M, Kapel CM (2005) Serological evidence of trichinellosis in local pigs of Nepal. Vet Parasitol 132: 155–157. 15978729 10.1016/j.vetpar.2005.05.046

[62] Sapkota B, Hörchner F, Srikitjakarn L, Kyule M, Baumann M, et al (2006) Seroprevalence of *Trichinella* in slaughter pigs in Kathmandu Valley, Nepal. Southeast Asian J Trop Med Public Health 37: 1078–1082. 17333757

[63] Karn SK, Horchner F, Srikitjakarn L, Baumann M, Nockler K (2008) Cross-sectional study of *Trichinella* spp in pigs in CDR, Nepal using pepsin digestion and ELISA serology. Southeast Asian J Trop Med Public Health 39: 795–799. 19058569

[64] Sethi B, Butola KS, Kumar Y, Mishra JP (2012) Multiple outbreaks of trichinellosis with high mortality rate. Trop Doct PMID 22472313. 10.1258/td.2012.12001E 23405010

[65] Rai SK, Uga S, Ono K, Nakanishi M, Shrestha HG, et al (1996) Seroepidemiological study of *Toxocara* infection in Nepal. Southeast Asian J Trop Med Public Health 27: 286–290. 9279991

[66] Shrestha S, Singh SD, Shrestha N, Shrestha R (2012) Clinical and laboratory profile of children with eosinophilia at Dhulikhel Hospital. Kathmandu Univ Med J (KUMJ) 38: 58–62. 10.3126/kumj.v10i2.7346 23132478

[67] Ghimire K (2002) Prevalence of intestinal parasites in humans and dogs of KMC particularly in Ward no 19, KMC [MSc dissertation]. Kathmandu: Tribhuvan University. 68 p.

[68] Karki S (2003) Prevalence of intestinal parasites in humans and dogs of KMC particularly in Ward no 20, KMC [MSc dissertation]. Kathmandu: Tribhuvan University. 69 p.

[69] Rai P (2002) Study on the fish parasites of exotic fished (rainbow trout and carp) and their control measures in different culture systems [MSc dissertation]. Kathmandu: Tribhuvan University. 44 p.

[70] Thapa RB (2000) Prevalence of intestinal helminth parasites in general and Taenia spp in detail, particularly in Bote and Darai communities of Vyash Muncipality-5, Kumaltari, Tanahun district of Nepal [MSc dissertation]. Kathmandu: Tribhuvan University. 91 p.

[71] Pandey K (2001) Prevalence of fasciolosis in buffaloes in relation to *Fasciola* larvae infection in *Lymnaea* snails in Dev Bhumi Baluwa VDC of Kavre district [MSc dissertation]. Kathmandu: Tribhuvan University. 58 p.

[72] Mahato SN, Harrison LJ (2005) Control of fasciolosis in stall-fed buffaloes by managing the feeding of rice straw. Trop Anim Health Prod 37: 285–291. 15934636 10.1007/s11250-005-3076-y

[73] Gurung B (2007) Prevalence of eggs of three trematode genera (*Fasciola* spp, *Dicrocoelium* spp and *Schistosoma* spp) in buffaloes of Satungal slaughter house, Kathmandu [MSc dissertation]. Kathmandu: Tribhuvan University. 52 p.

[74] Mukhia G (2007) A study on the intestinal helminth parasites of buffaloes brought to Satungal (KTM) for slaughter purpose [MSc dissertation]. Kathmandu: Tribhuvan University. 76 p.

[75] Shrestha A (2010) Prevalence of fascioliasis present in buffaloes of slaughter house in Kirtipur Municipality [MSc dissertation]. Kathmandu: Tribhuvan University. 85 p.

[76] Yong TS, Sim S, Lee J, Ohrr H, Kim MH, et al (2000) A small-scale survey on the status of intestinal parasite infections in rural villages in Nepal. Korean J Parasitol 38: 275–277. 11138322 10.3347/kjp.2000.38.4.275 PMC2721210

[77] Hamano S, Kobayashi S, Ogaki T, Koga M, Kawasaki M, et al (1999) A survey on helminthic infections in two rural communities in Nepal. Japanese Journal of Tropical Medicine and Hygiene 27: 511–515.

[78] Uga S, Rai SK, Kimura K, Ganesh R, Kimura D, et al (2004) Parasites detected from diarrheal stool samples collected in Nepal. Southeast Asian J Trop Med Public Health 35: 19–23. 15272739

[79] Kimura K, Rai SK, Rai G, Insisiengmay S, Kawabata M, et al (2005) Study on *Cyclospora cayetanensis* associated with diarrheal disease in Nepal and Loa PDR. Southeast Asian J Trop Med Public Health 36: 1371–1376. 16610636

[80] Shrestha S, Timila D, Karki U, Shrestha A, Bhandary S (2012) Clinical profile of children with moderate-to-severe eosinophilia presenting to a tertiary hospital in Nepal. Trop Doct 42: 232–234. 23405007 10.1258/td.2012.120284

[81] Kumari N, Kumar M, Rai A, Acharya A (2006) Intestinal trematode infection in North Bihar. JNMA J Nepal Med Assoc 45: 204–206. 17160098

[82] Rai S, Wadhwa V, Kharbanda P, Uppal B (2007) A case of poly-parasitism involving a trematode and four different nematodes in a migrant from Bihar. Indian J Med Microbiol 25: 62–63. 17377357 10.4103/0255-0857.31066

[83] Mahajan RK, Duggal S, Biswas NK, Duggal N, Hans C (2010) A finding of live *Fasciolopsis buski* in an ileostomy opening. J Infect Dev Ctries 4: 401–403. 20601794

[84] Prasad KN, Chawla S, Jain D, Pandey CM, Pal L, et al (2002) Human and porcine *Taenia solium* infection in rural north India. Trans R Soc Trop Med Hyg 96: 515–516. 12474478 10.1016/s0035-9203(02)90423-2

[85] Devleesschauwer B, Aryal A, Joshi DD, Rijal S, Sherchand JB, et al (2012) Epidemiology of *Taenia solium* in Nepal: is it influenced by the social characteristics of the population and the presence of *Taenia asiatica*? Trop Med Int Health 17: 1019–1022. 22643112 10.1111/j.1365-3156.2012.03017.x

[86] Nkouawa A, Sako Y, Nakao M, Nakaya K, Ito A (2009) Loop-mediated isothermal amplification method for differentiation and rapid detection of *Taenia* species. J Clin Microbiol 47: 168–174. 19005142 10.1128/JCM.01573-08 PMC2620829

[87] Joshi DD, Maharjan M, Johansen MV, Willingham AL, Gaihre YK, et al (2004) Taeniasis/cysticercosis situation in Nepal. Southeast Asian J Trop Med Public Health 35: 252–258.15691119

[88] Chakrabarti I, Gangopadhyay M, Bandopadhyay A, Das N (2012) A rare case of gangrenous appendicitis by eggs of *Taenia* species. Journal of Parasitic Diseases 1–3 doi:10.1007/s12639-012-0182-4 10.1007/s12639-012-0182-4 PMC3909584 24505193

[89] Criscione CD, Anderson JD, Raby K, Sudimack D, Subedi J, et al (2007) Microsatellite markers for the human nematode parasite *Ascaris lumbricoides*: development and assessment of utility. J Parasitol 93: 704–708. 17626368 10.1645/GE-1058R.1

[90] Criscione CD, Anderson JD, Sudimack D, Subedi J, Upadhayay RP, et al (2010) Landscape genetics reveals focal transmission of a human macroparasite. PLoS Negl Trop Dis 4: e665. 20421919 10.1371/journal.pntd.0000665 PMC2857643

[91] Navitsky RC, Dreyfuss ML, Shrestha J, Khatry SK, Stoltzfus RJ, et al (1998) *Ancylostoma duodenale* is responsible for hookworm infections among pregnant women in the rural plains of Nepal. J Parasitol 84: 647–651. 9645880

[92] Rai S, Shrestha H, Nakanishi M, Kubo T, Ono K, et al (1997) Hookworm infection recorded at an University Teaching Hospital in Kathmandu, Nepal over one decade period. Japanese Journal of Tropical Medicine and Hygiene 25: 81–84.

[93] Singh A, Janaki L, Petri WA Jr, Houpt ER (2009) *Giardia intestinalis* assemblages A and B infections in Nepal. Am J Trop Med Hyg 81: 538–539. 19706929 PMC3412867

[94] Wu Z, Nagano I, Boonmars T, Nakada T, Takahashi Y (2003) Intraspecies polymorphism of Cryptosporidium parvum revealed by PCR-restriction fragment length polymorphism (RFLP) and RFLP-single-strand conformational polymorphism analyses. Appl Environ Microbiol 69: 4720–4726. 12902263 10.1128/AEM.69.8.4720-4726.2003 PMC169079

[95] Wu Z, Nagano I, Boonmars T, Takahashi Y (2004) Further evidence that genotype I and genotype II of *Cryptosporidium parvum* are distinct. Tropical Medicine and Health 32: 5–14.

[96] Feng Y, Karna SR, Dearen TK, Singh DK, Adhikari LN, et al (2012) Common occurrence of a unique *Cryptosporidium ryanae* variant in zebu cattle and water buffaloes in the buffer zone of the Chitwan National Park, Nepal. Vet Parasitol 185: 309–314. 21996006 10.1016/j.vetpar.2011.09.025

[97] Yoshikawa H, Wu Z, Pandey K, Pandey BD, Sherchand JB, et al (2009) Molecular characterization of *Blastocystis* isolates from children and rhesus monkeys in Kathmandu, Nepal. Vet Parasitol 160: 295–300. 19136214 10.1016/j.vetpar.2008.11.029

[98] Lee IL, Tan TC, Tan PC, Nanthiney DR, Biraj MK, et al (2012) Predominance of *Blastocystis* sp. subtype 4 in rural communities, Nepal. Parasitol Res 110: 1553–1562. 22076050 10.1007/s00436-011-2665-0

[99] Lee LI, Chye TT, Karmacharya BM, Govind SK (2012) *Blastocystis* sp.: waterborne zoonotic organism, a possibility? Parasit Vectors 5: 130. 22741573 10.1186/1756-3305-5-130 PMC3434050

[100] Coyle CM, Varughese J, Weiss LM, Tanowitz HB (2012) *Blastocystis*: to treat or not to treat. Clin Infect Dis 54: 105–110. 22075794 10.1093/cid/cir810

[101] Heap BJ (1990) Cerebral cysticercosis as a common cause of epilepsy in Gurkhas in Hong Kong. J R Army Med Corps 136: 146–149. 2266527 10.1136/jramc-136-03-04

[102] Fegan D, Glennon J (1991) Epilepsy and disappearing lesions: adopting a wait and see policy. Bmj 302: 1402. 10.1136/bmj.302.6789.1402-d PMC1670092 1953856

[103] Prasad R, Taparia S (2004) Lateral sinus thrombosis with neurocysticercosis. Indian Pediatr 41: 1074–1075. 15523146

[104] Prasad R, Singh R, Joshi B (2005) Lateral sinus thrombosis in neurocysticercosis. Trop Doct 35: 182–183. 16105354 10.1258/0049475054620914

[105] Sundarka M (2005) Bilateral ptosis due to neurocysticercosis in the midbrain. Nepal Journal of Neuroscience 2: 137.

[106] Gurung G, Shrestha B, Shah TC (2005) Unusual presentation of neurocysticercosis: report of two cases. Nepal Journal of Neuroscience 2: 185–189.

[107] Mamkin I, Sood N, Ramanan SV (2007) *Taenia solium* neurocysticercosis. N Engl J Med 357: 1666–1667. 17942885 10.1056/NEJMc070837

[108] Shrestha R, Adhikari L (2009) Sequel of ring enhanced lesion on computerized tomographic scans of brain of patients presenting with seizure. Nepal Journal of Neuroscience 6: 12–14.

[109] Patnaik MM, Rajasingham R, Deshpande A, Parmar G, Stauffer W (2009) The Nepalese shepherd. J Travel Med 16: 68–71. 19192135 10.1111/j.1708-8305.2008.00275.x

[110] Azzopardi L, Quirk J (2012) An acquired source of seizures. Bmj 344: e2991. 22556049 10.1136/bmj.e2991

[111] KC I, Rana K, Joshi R, Mandal A, Bhhatarai S (2011) Multiple parenchymal neurocysticercosis: a case report. Medical Journal of Shree Birendra Hospital 10: 44–45.

[112] Rauniyar RK, Thakur SK, Panda A (2003) CT in the diagnosis of isolated cysticercal infestation of extraocular muscle. Clin Radiol 58: 154–156. 12623046 10.1053/crad.2002.1101

[113] Wong YC, Goh KY, Choo CT, Seah LL, Rootman J (2005) An unusual cause of acquired horizontal diplopia in a young adult. Br J Ophthalmol 89: 390–391. 15722329 10.1136/bjo.2004.052258 PMC1772552

[114] Shariq SM, Adhikari BP (2007) Managing cysticercosis in anterior chamber of eye: a case report. Kathmandu Univ Med J (KUMJ) 5: 240–242. 18604028

[115] Shrestha JB, Paudel P, Karmacharya PC (2008) Spontaneous extrusion of subconjunctival cysticercous cyst: a case report. Nepal Med Coll J 10: 139–140. 18828441

[116] Yadav SK, Winter I, Singh SK (2009) Management of intra-vitreal cysticercosis. Nepal J Ophthalmol 1: 143–145. 21141010 10.3126/nepjoph.v1i2.3692

[117] Amatya BM, Kimula Y (1999) Cysticercosis in Nepal: a histopathologic study of sixty-two cases. Am J Surg Pathol 23: 1276–1279. 10524530 10.1097/00000478-199910000-00014

[118] Sah SP, Jha PC, Gupta AK, Raj GA (2001) An incidental case of breast cysticercosis associated with fibroadenoma. Indian J Pathol Microbiol 44: 59–61. 12562000

[119] Smiti S, Sripathi H, Naik L (2003) Unusual location of *Cysticercus* lesions in soft tissue-report of three cases. Indian Journal of Radiology and Imaging 13: 157–158.

[120] Bhandary S, Singh R, Karki P, Sinha AK (2004) Cysticercosis of tongue–diagnostic dilemma. Pac Health Dialog 11: 87–88. 18181447

[121] Adhikari RC, Aryal G, Jha A, Pant AD, Sayami G (2007) Diagnosis of subcutaneous cysticercosis in fine needle aspirates: a study of 10 cases. Nepal Med Coll J 9: 234–238. 18298011

[122] Agarwal A, Murty O, Jain M (2009) Fine needle aspiration cytology in the diagnosis of cysticercosis cases. Asian Pacific Journal of Tropical Medicine 2: 49–53.

[123] Lakhey M, Hirachand S, Akhter J, Thapa B (2009) Cysticerci in palpable nodules diagnosed on fine needle aspiration cytology. JNMA J Nepal Med Assoc 48: 314–317. 21105557

[124] Sharma P, Neupane S, Shrestha M, Dwivedi R, Paudel K (2010) An ultrasonographic evaluation of solitary muscular and soft tissue cysticercosis. Kathmandu Univ Med J (KUMJ) 8: 257–260. 21209548 10.3126/kumj.v8i2.3571

[125] Rai B (2012) The cysticercosis in muscles is one of the causes of lumps in human. Health Renaissance 10: 160–161.

[126] Mukhida K, Sharma MR, Shilpakar SK (2004) Management of hydrocephalus with ventriculoperitoneal shunts: review of 274 cases. Nepal Journal of Neuroscience 1: 104–112.

[127] Karmacharya B, Kumar P (2012) A study on complications of ventriculoperitoneal shunt surgery in Bir Hospital, Kathmandu, Nepal. Nepal Journal of Medical Sciences 1: 119–122.

[128] Subedee A (2012) Evaluation of chronic headache by computed tomography: a retrospective study. Journal of Nobel Medical College 1: 64–71.

[129] Pant B, Devleesschauwer B, Shrestha P, Shrestha I, Praet N, et al (2011) Intraventricular *Taenia solium* neurocysticercosis: a report of three cases. JNMA J Nepal Med Assoc 51: 192–195. 22922900

[130] Rai SK, Shibata H, Sumi K, Kubota K, Hirai K, et al (1994) Seroepidemiological study of toxoplasmosis in two different geographical areas in Nepal. Southeast Asian J Trop Med Public Health 25: 479–484. 7777911

[131] Rai SK, Kubo T, Yano K, Shibata H, Sumi K, et al (1996) Seroepidemiological study of *Toxoplasma* infection in central and western regions in Nepal. Southeast Asian J Trop Med Public Health 27: 548–553. 9185267

[132] Rai SK, Shibata H, Sumi K, Rai G, Rai N, et al (1998) *Toxoplasma* antibody prevalence in Nepalese pregnant women and women with bad obstetric history. Southeast Asian J Trop Med Public Health 29: 739–743. 10772556

[133] Rai SK, Kubo T, Yano K, Shibata H, Sumi K, et al (1998) *Toxoplasma gondii* infection in Eastern Nepal-a seroepidemiological study. Journal of Infectious Diseases and Antimicrobial Agents 15: 105–109.

[134] Rai SK, Matsumura T, Ono K, Abe A, Hirai K, et al (1999) High *Toxoplasma* seroprevalence associated with meat eating habits of locals in Nepal. Asia Pac J Public Health 11: 89–93. 11195164 10.1177/101053959901100207

[135] Ray R (2010) Seroprevalence of IgM and IgG antibodies against the agents of TORCH infections among the patients visiting om hospital and research center [MSc dissertation]. Kathmandu: Tribhuvan University. 64 p.

[136] Imura SI, Ono K, Rai SK, Yanagida J-I, Ashitaka Y (2012) Seroprevalence of *Toxoplasma gondii* infection in Nepal. Bulletin of Kobe Tokiwa University 5: 9–14.

[137] Rai SK, Upadhyay MP, Shrestha HG (2003) *Toxoplasma* infection in selected patients in Kathmandu, Nepal. Nepal Med Coll J 5: 89–91. 15024774

[138] Kafle P (2004) Seroprevalence of TORCH in Nepalese women of childbearing age and evaluation of biochemical parameters [MSc dissertation]. Kathmandu: Tribhuvan University. 68 p.

[139] Lamichhane S, Malla S, Basnyat S, Khanal S, Dumre S, et al (2007) Seroprevalence of IgM antibodies against the agents of TORCH infections among the patients visiting National Public Health Laboratory, Teku Kathmandu. J Nepal Health Res Counc 5: 21–25.

[140] Kumari N, Morris N, Dutta R (2011) Is screening of TORCH worthwhile in women with bad obstetric history: an observation from eastern Nepal. J Health Popul Nutr 29: 77–80. 21528793 10.3329/jhpn.v29i1.7569 PMC3075056

[141] Graham LE, Flynn P, Patterson V (2002) Teleneuroradiology: a case from Nepal with clinical and educational benefit. J Telemed Telecare 8: 356–358. 12537924 10.1258/135763302320939257

[142] Ghimire P, Sapkota D, Manandhar SP (2004) Cryptosporidiosis: opportunistic infection in HIV/AIDS patients in Nepal. J Trop Med Parasitol 27: 7–10.

[143] Sapkota D, Ghimire P, Manandhar S (2004) Enteric parasitosis in patients with human immunodeficiency virus (HIV) Infection and acquired immunodeficiency syndrome (AIDS) in Nepal. J Nepal Health Res Counc 2: 9–13.

[144] Lamichhane G, Shah DN, Sharma S, Chaudhary M (2010) Ocular manifestations in HIV/AIDS cases in Nepal. Nepal J Ophthalmol 2: 45–50. 21141327 10.3126/nepjoph.v2i1.3704

[145] Rai SK, Sharma A, Shrestha RK, Pradhan P (2011) First case of congenital toxoplasmosis from Nepal. Nepal Med Coll J 13: 64–66. 21991707

[146] Maharjan M (1996) Incidence of zoonotic disease: Echinococcosis/hydatidosis in water buffaloes slaughtered for meat in Western part of Kathmandu [MSc dissertation]. Kathmandu: Tribhuvan University. 90 p.

[147] Joshi DD, Joshi AB, Joshi H (1997) Epidemiology of echinococcosis in Nepal. Southeast Asian J Trop Med Public Health 28 Suppl 1: 26–31. 9656344

[148] Khatri PS (2003) Study on prevalence of echinococcosis/hydatidosis in different livestock slaughtered in Banepa, Panauti and Dhulikhel municipalities of Kavre Palanchowk district and tis impact on public health [MSc dissertation]. Kathmandu: Tribhuvan University. 91 p.

[149] Manandhar S (2005) Occurence of echinococcosis/hydatidosis in slaughter buffaloes and *Echinococcus granulosus* in stray dogs in Kathmandu Valley, Nepal [MSc dissertation]. Chiang Mai and Berlin: Chiang Mai University and Freie Universität Berlin. 76 p.

[150] Sharma A (2008) Prevalence of cystic echinococcosis in buffaloes slaughtered in Kirtipur Municipality and its impact on public health [BSc dissertation]. Rampur: Tribhuvan University, Institute of Agriculture and Animal Sciences.

[151] Yadav N (2009) Investigation of diagnostic indicators of echinococcosis in slaughtering buffaloes of Chitwan District, Nepal [MSc dissertation]. Rampur: Tribhuvan University, Institute of Agriculture and Animal Sciences.

[152] Baronet D, Waltner-Toews D, Craig PS, Joshi DD (1994) *Echinococcus granulosus* infections in the dogs of Kathmandu, Nepal. Ann Trop Med Parasitol 88: 485–492. 7979638 10.1080/00034983.1994.11812895

[153] Sah SP, Agrawal CS, Khan IR, Rani S (2000) Hydatid cyst presenting as a breast lump. Southeast Asian J Trop Med Public Health 31: 185–186. 11023090

[154] Biswas R, Dhakal B, Das RN, Shetty KJ (2004) Resolving diagnostic uncertainty in initially poorly localizable fevers: a prospective study. Int J Clin Pract 58: 26–28. 14994967 10.1111/j.1368-5031.2004.0094.x

[155] Gupta D, Sharma MR, Shilpakar SK (2007) Giant hydatid cyst of brain. Nepal Journal of Neuroscience 4: 77.

[156] Khan JA, Devkota P, Acharya BM, Pradhan NM, Shreshtha S (2008) Bony hydatid disease of superior pubic ramus with extension into pelvis and proximal thigh. JNMA J Nepal Med Assoc 47: 139–141. 19079380

[157] Bhandari RS, Shrestha M, Shrestha GK, Mishra PR, Singh KP (2009) Abdominal lump: a diagnostic dilemma. JNMA J Nepal Med Assoc 48: 75–77. 19529065

[158] Mondal SK, Sengupta SG (2009) Hydatid cyst of radial bone. JNMA J Nepal Med Assoc 48: 321–323. 21105559

[159] Joshi A, Shrestha K, Shah L (2011) Infected hydatid cyst: various presentations. Postgraduate Medical Journal of NAMS 11: 59–61.

[160] Subedi PP, Niyaf A, Sharma MR, Shilpakar SK (2012) Surgical management of parasitic lesions of the central nervous system: our experience. Nepal Journal of Neuroscience 9: 21–28.

[161] Rauniyar R, Sharma U, Baboo S (2012) Isolated extra hepatic hydatid cyst of para spinal muscle - unusual presentation - a case report. Nepalese Journal of Radiology 2: 31–34.

[162] Bashyal K (2003) Case study of echinococcosis/hydatidosis in different hospitals of Kathmandu [MSc dissertation]. Kathmandu: Tribhuvan University. 60 p.

[163] Gautam BP (2009) Prevalence of cystic echinococcosis/hydatidosis in slaughtered buffaloes of Kathmandu metropolitan city and assessment of its impact on public health [BSc dissertation]. Rampur: Tribhuvan University, Institute of Agriculture and Animal Sciences. 55 p.

[164] Ito A, Okamoto M, Ishiguro T, Ma L, Suzuki H, et al (1998) Short report: An imported case of cystic echinococcosis in Japan diagnosed by imaging and serology with confirmation of *Echinococcus granulosus*-specific DNA sequences. Am J Trop Med Hyg 58: 790–792. 9660465 10.4269/ajtmh.1998.58.790

[165] Zhang LH, Joshi DD, McManus DP (2000) Three genotypes of *Echinococcus granulosus* identified in Nepal using mitochondrial DNA markers. Trans R Soc Trop Med Hyg 94: 258–260. 10974993 10.1016/s0035-9203(00)90313-4

[166] Joshi DD, Yamasaki H (2010) Histopathological and molecular confirmation of porcine cystic echinococcosis (CE)/hydatidosis in Nepal. Journal of Institute of Medicine 32: 54–58.

[167] Havelaar AH, Haagsma JA, Mangen MJ, Kemmeren JM, Verhoef LP, et al (2012) Disease burden of foodborne pathogens in the Netherlands, 2009. Int J Food Microbiol 156: 231–238. 22541392 10.1016/j.ijfoodmicro.2012.03.029

[168] World Bank (2000) Nepal: Operational Issues and Prioritization of Resources in the Health Sector. Kathmandu: The World Bank. 78 p. Available: http://www.healthnet.org.np/healthstat/worldbank/nepaloperational.pdf. Accessed 12 July 2013.

[169] Devleesschauwer B, Ale A, Duchateau L, Dorny P, Lake R, et al (2013) Understanding the burden of disease in Nepal: a call for local evidence. J Nepal Health Res Counc 11: 221–224. 24362617

[170] Praet N, Speybroeck N, Manzanedo R, Berkvens D, Nsame Nforninwe D, et al (2009) The disease burden of *Taenia solium* cysticercosis in Cameroon. PLoS Negl Trop Dis 3: e406. 19333365 10.1371/journal.pntd.0000406 PMC2656639

[171] Budke CM, Deplazes P, Torgerson PR (2006) Global socioeconomic impact of cystic echinococcosis. Emerg Infect Dis 12: 296–303. 16494758 10.3201/eid1202.050499 PMC3373106

[172] Havelaar AH, Kemmeren JM, Kortbeek LM (2007) Disease burden of congenital toxoplasmosis. Clin Infect Dis 44: 1467–1474. 17479945 10.1086/517511

[173] Kortbeek LM, Hofhuis A, Nijhuis CD, Havelaar AH (2009) Congenital toxoplasmosis and DALYs in the Netherlands. Mem Inst Oswaldo Cruz 104: 370–373. 19430666 10.1590/s0074-02762009000200034

[174] Bhattarai R, Budke CM, Carabin H, Proaño JV, Flores-Rivera J, et al (2012) Estimating the non-monetary burden of neurocysticercosis in Mexico. PLoS Negl Trop Dis 6: e1521. 22363827 10.1371/journal.pntd.0001521 PMC3283554

[175] Institute for Health Metrics and Evaluation (2013) GBD Uncertainty Visualization [database]. Available: http://www.healthmetricsandevaluation.org/gbd/visualizations/gbd-uncertainty-visualization. Accessed 28 October 2013.

