# Supplementary material for: The Burden of Parasitic Zoonoses in Nepal: A Systematic Review
Source: PLoS Negl Trop Dis. 2014 Jan 2;8(1):e2634. doi: 10.1371/journal.pntd.0002634 (PMC3879239; doi:10.1371/journal.pntd.0002634)
Supplement: Supporting Information S1 — Search strategy. (DOC) [file pntd.0002634.s002.doc]

# Supplementary material 1 — Search strategy

For each PZ, we constructed a search phrase consisting of the key word "Nepal" and any element of a list containing the name of the PZ, possible synonyms, and the name(s) of the causative parasite(s) (Table S1-1). For example, the PubMed search phrase for Alveolar echinococcosis was:

(Nepal AND ("Alveolar echinococcosis" OR "Alveolar hydatidosis" OR "Echinococcus multilocularis"))

Table S1-1. Search terms used for the considered parasitic zoonoses in the Nepalese burden of disease study (in alphabetical order)

| **Parasitic zoonosis** | **Synonyms and related terms** | **Pathogen name** |
| --- | --- | --- |
| Alveolar echinococcosis | Alveolar hydatidosis | *Echinococcus multilocularis* |
| Angiostrongylosis | Angiostrongyliasis | *Angiostrongylus* *cantonensis* |
| *Anisakidae* infections | – | *Anisakis*  *Pseudoterranova* |
| Capillariosis | Capillariasis | *Capillaria* |
| Cystic echinococcosis | Hydatid disease  Hydatidosis | *Echinococcus granulosus*  Hydatid cyst |
| Cysticercosis | Neurocysticercosis | *Taenia solium* |
| Diphyllobothriosis | Diphyllobothriasis  Bothriocephalosis  Bothriocephaliasis | *Diphyllobothrium*  *Bothriocephalus*  Broad tapeworm  Fish tapeworm |
| Dirofilariosis | Dirofilariasis | *Dirofilaria* |
| Foodborne trematodosis | Trematodiasis  Fasciolosis  Fascioliosis  Fasciolasis  Fascioliasis  Distomatosis  Fasciolopsosis  Fasciolopsiosis  Opisthorchosis  Opisthorchiasis  Clonorchiosis  Clonorchiasis  Paragonimosis  Paragonimiasis | Fluke  Trematode  *Fasciola*  *Fasciolopsis*  *Opisthorchis*  *Clonorchis*  *Paragonimus* |
| Gnathostomosis | Gnathostomiasis | *Gnathostoma* |
| Sparganosis | Spirometrosis | *Spirometra*  *Sparganum* |
| Taeniosis | Taeniasis  Tapeworm | *Taenia* |
| Toxocarosis | Toxocariasis  Toxocariosis  Larva migrans | *Toxocara* |
| Toxoplasmosis | TORCH | *Toxoplasma* |
| Trichinellosis | Trichinosis | *Trichinella* |
| Zoonotic intestinal protozoal infection | Protozoosis  Protozoasis  Giardiosis  Giardiasis  Cryptosporidiosis  Blastocystosis  Sarcocystosis | Protozoa  *Giardia*  *Cryptosporidium*  *Blastocystis*  *Sarcocystis* |
| Zoonotic schistosomosis | Schistosomiasis  Bilharziosis  Snail fever  Swimmers' itch | *Schistosoma*  *Bilharzia* |
| Zoonotic trypanosomosis | Trypanosomiasis  Chagas | *Trypanosoma cruzi* |
| Zoonotic leishmaniosis | Kala azar | *Leishmania* |
| Zoonotic intestinal helminth infection | Helminthosis  Helminthiasis  Ascarosis  Ascariasis  Ancylostomosis  Ancylostomiasis  Trichuriosis  Trichuriasis  Strongyloidosis  Strongyloidiasis | Helminth  *Ascaris*  *Ancylostoma*  Hookworm  *Trichuris*  *Strongyloides* |

Table S1-2 presents the major Nepalese journals and their websites that were included in the systematic review.

Table S1-2. Nepalese Journals

| **Journal** | **Website** |
| --- | --- |
| Journal of College of Medical Sciences Nepal | <http://cmsnepal.edu.np/> |
| Journal of Institute of Medicine | <http://www.jiom.com.np/> |
| Journal of Nepal Health Research Council | <http://www.jnhrc.com.np/> |
| Journal of Nepal Medical Association | <http://www.jnma.com.np/> |
| Journal of Nepal Paediatric Society | <http://www.nepjol.info/index.php/JNPS/issue/archive> |
| Kathmandu University Medical Journal | <http://www.kumj.com.np/> |
| Nepal Journal of Neuroscience | [http://www.neuroscience.org.np](http://www.neuroscience.org.np/) |
| Nepal Medical College Journal | <http://www.nmcth.edu/> |
