# Supplementary material for: The Burden of Parasitic Zoonoses in Nepal: A Systematic Review
Source: PLoS Negl Trop Dis. 2014 Jan 2;8(1):e2634. doi: 10.1371/journal.pntd.0002634 (PMC3879239; doi:10.1371/journal.pntd.0002634)
Supplement: Supporting Information S2 — Bayesian random-effects meta-analysis. (DOC) [file pntd.0002634.s003.doc]

# Supplementary material 2 — Bayesian random effects meta-analysis

The Bayesian random effects meta-analysis model for proportions was based on the following assumptions:

- The number of positive samples *x_i_* in each study results from a Binomial distribution with sample size *n_i_* and a study-specific true prevalence *θ_i_*;
- Each study-specific true prevalence (*θ_i_*) is the result of an overall true prevalence (*π*) and a random error. The variance of this random error equals the between-study variance, *τ²*. To prevent values outside (0,1) being sampled, the logit of *θ_i_* was modeled:


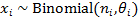


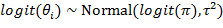


This model was implemented as follows:

model {

for (i in 1:N) {

x[i] ~ dbin(theta[i], n[i])

logit(theta[i]) <- theta.logit[i]

theta.logit[i] ~ dnorm(logit.pi, precision.theta)

}

tau <- sqrt(1 / precision.theta)

precision.theta ~ dgamma(1, 1)

logit(pi) <- logit.pi

logit.pi ~ dnorm(0, 0.001)

}

The model was run in Just Another Gibbs Sampler (JAGS 3.3.0; [1]), and its output was processed in R 3.0.1 through the R/JAGS interface provided by the rjags package [2,3]. For each model run, 6000 samples were generated, of which the first 1000 were discarded as burn-in. Two chains were run, starting from different, randomly chosen initial values. Based on these two chains, convergence was assessed visually using trace plots, and analytically using the Brooks-Gelman-Rubin statistic.

Subsequently, a Beta distribution was fitted to the MCMC simulations, using the fitdistrplus package in R [4].

# References

1. Plummer M (2003) JAGS: A program for analysis of Bayesian graphical models using Gibbs sampling. Proceedings of the 3rd International Workshop on Distributed Statistical Computing. Vienna, Austria: Technische Universität Wien. 10 p.

2. R Core Team (2013) R: A language and environment for statistical computing. Vienna, Austria: R Foundation for Statistical Computing. Available: <http://www.R-project.org/>.

3. Plummer M (2013) rjags: Bayesian graphical models using MCMC. R package version 3-10. Available: <http://CRAN.R-project.org/package=rjags>.

4. Delignette-Muller ML, Pouillot R, Denis JB, Dutang C (2013) fitdistrplus: help to fit of a parametric distribution to non-censored or censored data. R package version 1.0-1. Available: <http://CRAN.R-project.org/package=fitdistrplus>.
