# Supplementary material for: The Burden of Parasitic Zoonoses in Nepal: A Systematic Review
Source: PLoS Negl Trop Dis. 2014 Jan 2;8(1):e2634. doi: 10.1371/journal.pntd.0002634 (PMC3879239; doi:10.1371/journal.pntd.0002634)
Supplement: Supporting Information S3 — Quantitative burden assessment. (DOCX) [file pntd.0002634.s004.docx]

# Supplementary material 3 — Quantitative burden assessment

Table of Contents

[1. Taeniosis 2](#_Toc351453625)

[2. Intestinal Helminths 4](#_Toc351453626)

[3. Intestinal Protozoa 10](#_Toc351453627)

[4. Neurocysticercosis 16](#_Toc351453628)

[5. Toxoplasmosis 19](#_Toc351453629)

[6. Cystic echinococcosis 23](#_Toc351453630)

[References 26](#_Toc351453631)

# 1. Taeniosis

In total, 24 datasets could be retrieved from 20 documents (Table S3-1). Of these, 15 datasets were from community-based studies and 8 from hospital-based studies. One dataset included both community and clinical samples, and was therefore not included in the meta-analyses. Since mostly positive studies are included, these estimates presumably overestimate the average taeniosis prevalence.

Table S3-1. Raw data of retrieved *Taenia* spp. prevalence studies. Populations are given if the study targeted specific castes or ethnic groups. The diagnostic method was either direct smear (stained or unstained), sedimentation (e.g., Formol-Ether), flotation (e.g., sucrose), sedimentation and flotation, or self-detection.

| **Reference, First Author, Publication Year** | **District (Population)** | **Study Period** | **Diagnostic Method** | **Sample Size** | **Number Positive (%)** |
| --- | --- | --- | --- | --- | --- |
| ***Community-based studies*** | | | | | |
| [1] Gaihre 2000 | Syangja (Sarki) | 1999-2000 | Direct smear | 58 | 16 (27.6) |
| [1] Gaihre 2000 | Syangja (Magar) | 1999-2000 | Direct smear | 122 | 61 (50.0) |
| [2] Thapa 2000 | Tanahun (Bote) | 1999 | Direct smear | 62 | 19 (30.6) |
| [2] Thapa 2000 | Tanahun (Darai) | 1999 | Direct smear | 90 | 9 (10.0) |
| [3] Pradhan 2001 | Nawalparasi (Magar, Majhi) | 2000-2001 | Flotation | 240 | 2 (0.8) |
| [4] Karki 2003 | Palpa (Magar) | 2002-2003 | Direct smear | 157 | 13 (8.3) |
| [5] Karki 2003 | Kathmandu | 2001-2002 | Direct smear | 44 | 0 (0.0) |
| [6] Parajuli 2003 | Chitwan (Mushar) | 2002-2003 | Direct smear | 183 | 3 (1.6) |
| [7] Sharma 2007 | Bardiya (Tharu) | 2006-2007 | Sedimentation/Flotation | 257 | 4 (1.6) |
| [8] Shrestha 2007 | Kathmandu Valley | 2006-2007 | Sedimentation/Flotation | 315 | 1 (0.3) |
| [9] Devleesschauwer 2012 | Morang (Dum) | 2009-2011 | Self-detection | 524 | 71 (13.5) |
| [9] Devleesschauwer 2012 | Morang (other) | 2009-2011 | Self-detection | 1012 | 0 (0.0) |
| [10,11] Gyawali 2003, 2012 | Nawalparasi (Kumal) | 2000-2001 | Flotation | 149 | 3 (2.0) |
| [12] Lee 2012 | Kavre, Sindhupalchowk | 2009 | Sedimentation | 241 | 1 (0.4) |
| [13] Sah 2012 | Sunsari | 2007-2008 | Direct smear | 935 | 51 (5.5) |
| ***Hospital-based studies*** | | | | | |
| [14] Joshi 2001 | Sunsari | 1994-1996 | NA | 4445 | 4 (0.1) |
| [5] Karki 2003 | Kathmandu | 2001-2002 | Direct smear | 173 | 2 (1.2) |
| [15] Das 2006 | Kaski | 2000-2002 | Sedimentation | 5236 | 38 (0.7) |
| [16] Sherchand 2006 | Kathmandu | 2005 | Direct smear | 681 | 16 (2.3) |
| [17] Maharjan 2009 | Kathmandu | 2007 | Direct smear | 300 | 1 (0.3) |
| [18] Shakya 2009 | Parsa | 2006-2008 | Direct smear | 2221 | 12 (0.5) |
| [19] Khanal 2011 | Dang | 2010-2011 | Direct smear | 210 | 1 (0.5) |
| [20] Thapa 2011 | Kathmandu | 2009-2010 | Sedimentation | 4176 | 5 (0.1) |
| ***Mixed community/hospital-based studies*** | | | | | |
| [21] Ghimire 2002 | Kathmandu | 2000-2001 | Direct smear | 211 | 3 (1.4) |

# 2. Intestinal Helminths

In total, 67 datasets could be retrieved from 65 documents (Table S3-2). Of these, 37 datasets were from community-based studies, 25 from hospital-based studies, and 4 from studies on HIV-AIDS patients. One dataset included both community and clinical samples, and was therefore not included in the meta-analyses.

Table S3-2. Raw data of retrieved intestinal helminth prevalence studies. Populations are given if the study targeted specific castes or ethnic groups. The diagnostic method was either direct smear (stained or unstained), Kato-Katz, sedimentation (e.g., Formol-Ether), flotation (e.g., sucrose) or sedimentation and flotation.

| **Reference, First Author, Publication Year** | **District (Population)** | **Study Period** | **Diagnostic Method** | **Sample Size** | **Number Positive (%)** | | |
| --- | --- | --- | --- | --- | --- | --- | --- |
|  |  |  |  |  | *Ascaris* spp. | *Trichuris* spp. | Hookworm |
| ***Community-based studies*** | | | | | | | |
| [22] Shrestha 2000 | Kathmandu, Bhaktapur | 1998-1999 | Direct smear | 357 | 123 (34.5) | 21 (5.9) | 8 (2.2) |
| [2] Thapa 2000 | Tanahun (Bote, Darai) | 1999 | Direct smear | 152 | 33 (21.7) | 11 (7.2) | 30 (19.7) |
| [23] Yong 2000 | Chitwan | 1999 | Sedimentation | 300 | 5 (1.7) | 9 (3.0) | 39 (13.0) |
| [3] Pradhan 2001 | Nawalparasi (Magar, Majhi) | 2000-2001 | Flotation | 240 | 44 (18.3) | 12 (5.0) | 12 (5.0) |
| [24] Rijal 2001 | Chitwan | NA | Direct smear | 182 | 5 (2.7) | 1 (0.5) | 27 (14.8) |
| [25] Goto 2002 | Kathmandu | 1999-2000 | Direct smear | 173 | 102 (59.0) | 64 (37.0) | 16 (9.2) |
| [26] Williams-Blangero 2002 | Dolakha (Jirel) | 1995-1996 | Kato-Katz | 444 | 121 (27.3) | NA | NA |
| [27] Chaudhary 2003 | Kathmandu | 2002-2003 | Sedimentation | 306 | 102 (33.3) | 53 (17.3) | 4 (1.3) |
| [5] Karki 2003 | Kathmandu | 2001-2002 | Direct smear | 44 | 6 (13.6) | 5 (11.4) | 1 (2.3) |
| [4] Karki 2003 | Palpa (Magar) | 2002-2003 | Direct smear | 157 | 79 (50.3) | 27 (17.2) | 38 (24.2) |
| [28] Moffat 2003 | Kathmandu | 1995 | Direct smear | 71 | 7 (9.9) | 1 (1.4) | 0 (0.0) |
| [6] Parajuli 2003 | Chitwan (Mushar) | 2002-2003 | Direct smear | 183 | 88 (48.1) | 41 (22.4) | 64 (35.0) |
| [29] Rai 2003 | Kathmandu | 2002 | Sedimentation | 340 | 53 (15.6) | 129 (37.9) | 89 (26.2) |
| [30] Sherchand 2003 | Kathmandu | 2003 | Sedimentation | 176 | 62 (35.2) | 97 (55.1) | 73 (41.5) |
| [31] Malla 2004 | Sarlahi | 2004 | Direct smear | 225 | 32 (14.2) | 19 (8.4) | 10 (4.4) |
| [32] Sharma 2004 | Kathmandu Valley | NA | Sedimentation | 533 | 87 (16.3) | 218 (40.9) | 149 (28.0) |
| [33] Williams-Blangero 2004 | Dolakha (Jirel) | NA | Kato-Katz | 367 | 66 (18.0) | 50 (13.6) | 173 (47.1) |
| [34] Ghimire 2005 | Kathmandu, Chitwan | 2005 | Sedimentation | 400 | 41 (10.2) | 20 (5.0) | 15 (3.8) |
| [35] Kunwar 2006 | Dolakha | NA | NA | 478 | 97 (20.3) | 13 (2.7) | 253 (52.9) |
| [36] Majhi Tharu 2006 | Gorkha (Chepang) | 2004 | Direct smear | 225 | 172 (76.4) | 139 (61.8) | 156 (69.3) |
| [37] Shakya 2006 | Kathmandu Valley | 2005-2006 | Sedimentation/Flotation | 235 | 8 (3.4) | 52 (22.1) | 11 (4.7) |
| [38] Adhikari 2007 | Kathmandu Valley | 2006 | Sedimentation | 309 | 32 (10.4) | 67 (21.7) | 15 (4.9) |
| [39] Albonico 2007 | Syangja | 2004 | Kato-Katz | 1277 | 432 (33.8) | 1016 (79.6) | 624 (48.9) |
| [40] Jamarkattel 2007 | Kaski (Jalari, Kumal) | 2003 | Direct smear | 236 | 41 (17.4) | 7 (3.0) | NA |
| [7] Sharma 2007 | Bardiya (Tharu) | 2006-2007 | Sedimentation/Flotation | 257 | 12 (4.7) | 9 (3.5) | 34 (13.2) |
| [41] Shrestha 2007 | Kathmandu Valley | 2005-2006 | Sedimentation | 188 | 37 (19.7) | 66 (35.1) | 2 (1.1) |
| [8] Shrestha 2007 | Bhaktapur | 2006-2007 | Sedimentation/Flotation | 315 | 62 (19.7) | 116 (36.8) | 32 (10.2) |
| [42] Rai 2008 | Dhading, Ramechhap, Sindhupalchowk | 2006 | Sedimentation | 221 | 53 (24.0) | 36 (16.3) | 69 (31.2) |
| [43] Albonico 2009 | Dhading | 2006 | Kato-Katz | 1325 | 451 (34.0) | 636 (48.0) | 596 (45.0) |
| [44] Gyawali 2009 | Sunsari | 2007-2008 | Sedimentation | 182 | 6 (3.3) | 0 (0.0) | 3 (1.6) |
| [45] Parajuli 2009 | Parsa (Mushar, Tharu) | 2006 | Direct smear | 95 | 25 (26.3) | 6 (6.3) | 9 (9.5) |
| [46] Bhandari 2011 | Kavre | 2008-2009 | Sedimentation | 360 | 56 (15.6) | 66 (18.3) | 43 (11.9) |
| [47] Thapa Magar 2011 | Kathmandu Valley | 2008 | Sedimentation | 279 | 10 (3.6) | 12 (4.3) | 6 (2.2) |
| [10] Gyawali 2012 | Nawalparasi (Kumal) | 2000-2001 | Flotation | 149 | 24 (16.1) | 5 (3.4) | 46 (30.9) |
| [12] Lee 2012 | Kavre, Sindhupalchowk | 2009 | Sedimentation | 241 | 3 (1.2) | 26 (10.8) | 5 (2.1) |
| [48] Shakya 2012 | Parsa | 2008 | Direct smear | 165 | 7 (4.2) | 0 (0.0) | 1 (0.6) |
| [49] Shrestha 2012 | Baglung | 2010-2011 | Sedimentation | 260 | 6 (2.3) | 13 (5.0) | 7 (2.7) |
| ***Hospital-based studies*** | | | | | | | |
| [50] Chand 2000 | Kathmandu | 1999 | Sedimentation/Flotation | 272 | 18 (6.6) | 3 (1.1) | 9 (3.3) |
| [22] Shrestha 2000 | Kathmandu | 1998-1999 | Direct smear | 515 | 46 (8.9) | 22 (4.3) | 5 (1.0) |
| [51] Shrestha 2001 | Kathmandu | 1998 | Direct smear | 341 | 30 (8.8) | 17 (5.0) | 26 (7.6) |
| [52] Pandey 2002 | Kathmandu | 2001 | Sedimentation | 181 | 20 (11.0) | 50 (27.6) | 23 (12.7) |
| [27] Chaudhary 2003 | Kathmandu | 2002-2003 | Sedimentation | 194 | 17 (8.8) | 3 (1.5) | 5 (2.6) |
| [5] Karki 2003 | Kathmandu | 2001-2002 | Direct smear | 173 | 39 (22.5) | 15 (8.7) | 26 (15.0) |
| [53] Rai 2004 | Kathmandu | 2002 | Direct smear | 301 | 10 (3.3) | 2 (0.7) | 3 (1.0) |
| [54] Uga 2004 | Kathmandu | 1999-2001 | Sedimentation | 396 | 48 (12.1) | 104 (26.3) | 54 (13.6) |
| [55] Khadka 2005 | Kathmandu | 2004 | Direct smear | 311 | 16 (5.1) | 2 (0.6) | 1 (0.3) |
| [15] Das 2006 | Kaski | 2000-2002 | Sedimentation | 5236 | 116 (2.2) | 0 (0.0) | 68 (1.3) |
| [56] Lama 2006 | Kathmandu | 2005 | Direct smear | 340 | 10 (2.9) | 9 (2.6) | 5 (1.5) |
| [57] Lama 2007;  [58] Sherchand 2009 | Kathmandu | 2005 | Flotation | 440 | 15 (3.4) | 11 (2.5) | 9 (2.0) |
| [59] Mukhopadhyay 2007 | Kaski (persistent diarrhea) | 1998-2004 | Direct smear | 253 | 25 (9.9) | 18 (7.1) | 17 (6.7) |
| [41] Shrestha 2007 | Kathmandu Valley | 2005-2006 | Sedimentation | 1316 | 15 (1.1) | 4 (0.3) | 11 (0.8) |
| [60] Kandel 2008 | Kathmandu | 2005 | Direct smear | 278 | 50 (18.0) | 3 (1.1) | 6 (2.2) |
| [61] Shrestha 2008 | Kathmandu | 2005 | Direct smear | 340 | 2 (0.6) | 1 (0.3) | 0 (0.0) |
| [62] Tandukar 2008 | Kathmandu | 2006-2007 | Direct smear | 607 | 8 (1.3) | 5 (0.8) | 3 (0.5) |
| [17] Maharjan 2009 | Kathmandu | 2007 | Direct smear | 300 | 3 (1.0) | 2 (0.7) | 1 (0.3) |
| [63] Pokharel 2009 | Kathmandu | 2007 | Direct smear | 500 | 7 (1.4) | 5 (1.0) | 2 (0.4) |
| [18] Shakya 2009 | Parsa | 2006-2008 | Direct smear | 2221 | 149 (6.7) | 0 (0.0) | 86 (3.9) |
| [64] Basnet 2010 | Kathmandu | 2007 | Sedimentation/Flotation | 100 | 5 (5.0) | 3 (3.0) | 7 (7.0) |
| [65] Amatya 2011 | Sunsari | 2007-2008 | Sedimentation | 863 | 0 (0.0) | 1 (0.1) | 0 (0.0) |
| [19] Khanal 2011 | Dang | 2010-2011 | Direct smear | 210 | 14 (6.7) | 2 (1.0) | 6 (2.9) |
| [20] Thapa 2011 | Kathmandu | 2009-2010 | Sedimentation | 4176 | 11 (0.3) | 10 (0.2) | 16 (0.4) |
| [66] Ansari 2012 | Kathmandu | 2011 | Flotation | 525 | 3 (0.6) | 0 (0.0) | 0 (0.0) |
| ***HIV-aids patients*** | | | | | | | |
| [67] Sapkota 2003;  [68] Sapkota 2004 | Kathmandu, Jhapa | 2002-2003 | Sedimentation/Flotation | 75 | 0 (0.0) | 2 (2.7) | 1 (1.3) |
| [69] Adhikari 2006 | Kathmandu | 2005 | Sedimentation/Flotation | 196 | 11 (5.6) | 32 (16.3) | 29 (14.8) |
| [64] Basnet 2010 | Kathmandu | 2007 | Sedimentation/Flotation | 200 | 7 (3.5) | 5 (2.5) | 0 (0.0) |
| [70] Amatya 2011 | Sunsari | 2007-2008 | Sedimentation | 122 | 0 (0.0) | 0 (0.0) | 3 (2.5) |
| ***Mixed…*** | | | | | | | |
| [21] Ghimire 2002 | Kathmandu | 2000-2001 | Direct smear | 211 | 41 (19.4) | 22 (10.4) | 30 (14.2) |

# 3. Intestinal Protozoa

In total, 69 datasets could be retrieved from 64 documents (Table S3-3). Of these, 29 datasets were from community-based studies, 31 from hospital-based studies, and 8 from studies on HIV-AIDS patients. One dataset included both community and clinical samples, and was therefore not included in the meta-analyses.

Table S3-3. Raw data of retrieved intestinal protozoa prevalence studies. Populations are given if the study targeted specific castes or ethnic groups. The diagnostic method was either direct smear (stained or unstained), sedimentation (e.g., Formol-Ether), flotation (e.g., sucrose) or sedimentation and flotation, possibly combined with a protozoa-specific stain (e.g., Ziehl-Neelsen), fluorescence assay, or PCR.

| **Reference, First Author, Publication Year** | **District (Population)** | **Study Period** | **Diagnostic Method** | **Sample Size** | **Number Positive (%)** | | |
| --- | --- | --- | --- | --- | --- | --- | --- |
|  |  |  |  |  | *Giardia* spp. | *Cryptosporidium* spp. | *Blastocystis hominis* |
| ***Community-based studies*** | | | | | | | |
| [22] Shrestha 2000 | Kathmandu, Bhaktapur | 1998-1999 | Direct smear | 357 | 34 (9.5) | NA | NA |
| [23] Yong 2000 | Chitwan | 1999 | Sedimentation | 300 | 41 (13.7) | NA | NA |
| [24] Rijal 2001 | Chitwan | NA | Direct smear | 182 | 33 (18.1) | NA | NA |
| [25] Goto 2002 | Kathmandu | 1999-2000 | Direct smear | 173 | 24 (13.9) | NA | NA |
| [71] Shariff 2002 | Sunsari (non-diarrhea clinical) | 1999-2000 | Direct smear + Stain | 50 | 1 (2.0) | 0 (0.0) | NA |
| [27] Chaudhary 2003 | Kathmandu | 2002-2003 | Sedimentation + Stain | 306 | 38 (12.4) | 4 (1.3) | NA |
| [5] Karki 2003 | Kathmandu | 2001-2002 | Direct smear | 44 | 0 (0.0) | NA | NA |
| [28] Moffat 2003 | Kathmandu | 1995 | Direct smear | 71 | 17 (23.9) | NA | NA |
| [6] Parajuli 2003 | Chitwan (Mushar) | 2002-2003 | Direct smear | 183 | 14 (7.7) | NA | NA |
| [29] Rai 2003 | Kathmandu | 2002 | Sedimentation | 340 | 31 (9.1) | NA | NA |
| [30] Sherchand 2003 | Kathmandu | 2003 | Sedimentation + Stain | 176 | 71 (40.3) | 3 (1.7) | NA |
| [31] Malla 2004 | Sarlahi | 2004 | Direct smear | 225 | 21 (9.3) | NA | NA |
| [32] Sharma 2004 | Kathmandu Valley | NA | Sedimentation | 533 | 36 (6.8) | NA | NA |
| [33] Williams-Blangero 2004 | Dolakha (Jirel) | NA | Kato-Katz | 367 | 72 (19.6) | NA | NA |
| [72] Ghimire 2005 | Kathmandu (non-diarrhea clinical) | 2002-2004 | Sedimentation + Stain | 2643 | NA | 53 (2.0) | NA |
| [34] Ghimire 2005 | Kathmandu, Chitwan | 2005 | Sedimentation + Stain | 400 | 33 (8.2) | 4 (1.0) | NA |
| [73] Majhi Tharu 2006 | Gorkha (Chepang) | 2004 | Direct smear | 225 | 25 (11.1) | 9 (4.0) | NA |
| [37] Shakya 2006 | Kathmandu Valley | 2005-2006 | Sedimentation/Flotation + Stain | 235 | 2 (0.9) | 2 (0.9) | 7 (3.0) |
| [40] Jamarkattel 2007 | Kaski (Jalari, Kumal) | 2003 | Direct smear | 236 | 33 (14.0) | NA | NA |
| [59] Mukhopadhyay 2007 | Kaski (non-diarrhea clinical) | 1998-2004 | Direct smear + Stain | 100 | 8 (8.0) | 0 (0.0) | NA |
| [7] Sharma 2007 | Bardiya (Tharu) | 2006-2007 | Sedimentation/Flotation + Stain | 257 | 37 (14.4) | 0 (0.0) | 12 (4.7) |
| [8] Shrestha 2007 | Bhaktapur | 2006-2007 | Sedimentation/Flotation + Stain | 315 | 36 (11.4) | 0 (0.0) | 25 (7.9) |
| [42] Rai 2008 | Dhading, Ramechhap, Sindhupalchowk | 2006 | Sedimentation | 221 | 1 (0.5) | NA | NA |
| [44] Gyawali 2009 | Sunsari | 2007-2008 | Sedimentation | 182 | 23 (12.6) | NA | NA |
| [46] Bhandari 2011 | Kavre | 2008-2009 | Sedimentation | 360 | 21 (5.8) | NA | NA |
| [47] Thapa Magar 2011 | Kathmandu Valley | 2008 | Sedimentation | 279 | 48 (17.2) | 0 (0.0) | 6 (2.2) |
| [12] Lee 2012 | Kavre, Sindhupalchowk | 2009 | PCR (*Blastocystis*), Sedimentation + Stain (others) | 241 | 13 (5.4) | 1 (0.4) | 63 (26.1) |
| [48] Shakya 2012 | Parsa | 2008 | Direct smear | 165 | 5 (3.0) | NA | NA |
| [49] Shrestha 2012 | Baglung | 2010-2011 | Sedimentation | 260 | 15 (5.8) | NA | NA |
| ***Hospital-based studies*** | | | | | | | |
| [50] Chand 2000 | Kathmandu | 1999 | Sedimentation/Flotation + Stain | 272 | 16 (5.9) | 8 (2.9) | 0 (0.0) |
| [22] Shrestha 2000 | Kathmandu | 1998-1999 | Direct smear | 515 | 29 (5.6) | NA | NA |
| [74] Ono 2001 | Kathmandu | 1996-1997 | Flotation + Fluorescence | 334 | 15 (4.5) | 8 (2.4) | 5 (1.5) |
| [51] Shrestha 2001 | Kathmandu | 1998 | Direct smear | 341 | 12 (3.5) | NA | NA |
| [52] Pandey 2002 | Kathmandu | 2001 | Sedimentation + Stain | 181 | 14 (7.7) | 14 (7.7) | 14 (7.7) |
| [71] Shariff 2002 | Sunsari | 1999-2000 | Direct smear + Stain | 160 | 0 (0.0) | 9 (5.6) | NA |
| [27] Chaudhary 2003 | Kathmandu | 2002-2003 | Sedimentation + Stain | 194 | 19 (9.8) | 10 (5.2) | 0 (0.0) |
| [5] Karki 2003 | Kathmandu | 2001-2002 | Direct smear | 173 | 14 (8.1) | NA | NA |
| [53] Rai 2004;  [75] Rai 2005 | Kathmandu | 2002 | Direct smear | 301 | 36 (12.0) | 4 (1.3) | NA |
| [76] Dhakal 2004 | Kathmandu | 2002 | Sedimentation/Flotation + Stain | 460 | NA | 48 (10.4) | NA |
| [54] Uga 2004 | Kathmandu | 1999-2001 | Sedimentation | 396 | 40 (10.1) | 1 (0.3) | NA |
| [72] Ghimire 2005 | Kathmandu | 2002-2004 | Sedimentation + Stain | 6357 | NA | 964 (15.2) | NA |
| [55] Khadka 2005 | Kathmandu | 2004 | Direct smear | 311 | 24 (7.7) | NA | NA |
| [15] Das 2006 | Kaski | 2000-2002 | Sedimentation | 5236 | 1098 (21.0) | NA | NA |
| [56] Lama 2006 | Kathmandu | 2005 | Direct smear | 340 | 33 (9.7) | NA | NA |
| [57] Lama 2007 | Kathmandu | 2005 | Flotation + Stain | 440 | 21 (4.8) | 4 (0.9) | NA |
| [59] Mukhopadhyay 2007 | Kaski (acute diarrhea) | 1998-2004 | Direct smear + Stain | 100 | 4 (4.0) | 0 (0.0) | NA |
| [59] Mukhopadhyay 2007 | Kaski (persistent diarrhea) | 1998-2004 | Direct smear + Stain | 253 | 61 (24.1) | 2 (0.8) | NA |
| [60] Kandel 2008 | Kathmandu | 2005 | Direct smear | 278 | 59 (21.2) | NA | NA |
| [61] Shrestha 2008 | Kathmandu | 2005 | Direct smear | 340 | 6 (1.8) | NA | NA |
| [62] Tandukar 2008 | Kathmandu | 2006-2007 | Direct smear | 607 | 8 (1.3) | 5 (0.8) | 3 (0.5) |
| [17] Maharjan 2009 | Kathmandu | 2007 | Direct smear | 300 | 35 (11.7) | NA | NA |
| [63] Pokharel 2009 | Kathmandu | 2007 | Direct smear | 500 | 32 (6.4) | NA | NA |
| [18] Shakya 2009 | Parsa | 2006-2008 | Direct smear | 2221 | 26 (1.2) | NA | NA |
| [77] Singh 2009 | Kathmandu | NA | Direct smear | 1096 | 45 (4.1) | NA | NA |
| [78] Yoshikawa 2009 | Kathmandu | 2003 | Direct smear? | 82 | NA | NA | 21 (25.6) |
| [64] Basnet 2010 | Kathmandu | 2007 | Sedimentation/ Flotation + Stain | 100 | 6 (6.0) | 1 (1.0) | NA |
| [65] Amatya 2011 | Sunsari | 2007-2008 | Sedimentation + Stain | 863 | 33 (3.8) | 36 (4.2) | NA |
| [20] Thapa 2011 | Kathmandu | 2009-2010 | Sedimentation | 4176 | 252 (6.0) | NA | NA |
| [66] Ansari 2012 | Kathmandu | 2011 | Flotation + Stain | 525 | 18 (3.4) | 0 (0.0) | NA |
| [79] Sherchand 2012 | Kathmandu | 2009-2010 | Sedimentation + Stain | 1721 | 23 (1.3) | 3 (0.2) | 2 (0.1) |
| ***HIV-aids patients*** | | | | | | | |
| [67] Sapkota 2003;  [80] Ghimire 2004;  [68] Sapkota 2004 | Kathmandu, Jhapa | 2002-2003 | Sedimentation/Flotation + Stain | 75 | 5 (6.7) | 8 (10.7) | NA |
| [81] Das 2005 | Kaski | 2001-2002 | NA | 74 | NA | 6 (8.1) | NA |
| [69] Adhikari 2006 | Kathmandu | 2005 | Sedimentation/Flotation + Stain | 196 | 2 (1.0) | 2 (1.0) | 1 (0.5) |
| [82] Mishra 2009 | Kaski | 2004-2005 | Direct smear + Stain | 53 | NA | 6 (11.3) | NA |
| [64] Basnet 2010 | Kathmandu | 2007 | Sedimentation/ Flotation + Stain | 200 | 14 (7.0) | 13 (6.5) | NA |
| [83] Sharma 2010 | Kathmandu Valley | 2007-2008 | Direct smear + Stain | 150 | NA | 29 (19.3) | NA |
| [70] Amatya 2011 | Sunsari | 2007-2008 | Sedimentation + Stain | 122 | 7 (5.7) | 5 (4.1) | 2 (1.6) |
| [84] Sherchan 2012 | Kathmandu | 2010-2011 | Sedimentation + Stain | 146 | 14 (9.6) | 4 (2.7) | 9 (6.2) |
| ***Mixed …*** | | | | | | | |
| [21] Ghimire 2002 | Kathmandu | 2000-2001 | Direct smear | 211 | 14 (6.6) | NA | NA |

# 4. Neurocysticercosis

The burden assessment for neurocysticercosis (NCC) is based on Praet et al. [85]. Figure S3-1 shows the underlying computational disease model. Table S3-6 summarizes the applied DALY parameters.

Epilepsy

incidence

NCC-associated

epilepsy

Death

Figure S3-1. Computational disease model for NCC. Rectangles with sharp corners represent incidences, those with rounded corners conditional probabilities. Green shapes contribute YLDs, red shapes contribute YLLs.

To model the epilepsy incidence, we divided the epilepsy prevalence by its mean duration. Two sources were identified for epilepsy prevalence (Table S3-4), which were used as the minimum and maximum in a Uniform distribution. The mean epilepsy duration was based on Praet et al. [85].

Table S3-4. Retrieved epilepsy prevalence data

| **Reference, First Author, Publication Year** | **District** | **Study Period** | **Diagnostic Method** | **Sample Size** | **Number Positive (‰)** |
| --- | --- | --- | --- | --- | --- |
| [86] UNICEF 2001 | National sample | 1999-2000 | Questionnaire | 75,994 | 189 (2.5) |
| [87] Rajbhandari 2004 | Morang | NA | Questionnaire | 4636 | 34 (7.3) |

Different studies were identified that estimated the proportion of epilepsy cases associated with NCC (Table S3-5). Bayesian random effects meta-analysis was applied to these data to obtain a single Beta distribution (Supplementary material S2).

Table S3-5. Retrieved proportions of epilepsy cases associated with neurocysticercosis

| **Reference, First Author, Publication Year** | **District** | **Study Period** | **Diagnostic Method** | **Sample Size** | **Number Positive (%)** |
| --- | --- | --- | --- | --- | --- |
| [87] Rajbhandari 2004 | Birendra Military Hospital | 2000 | EEG, MRI | 300 | 141 (47.0) |
| [88] Neupane 2006 | Kathmandu | 2002 | CT, MRI | 200 | 46 (23.0) |
| [89] Chaudhary 2006 | Lalitpur | 2001-2005 | CT, MRI | 543 | 39 (7.2) |
| [90] Piryani 2007 | Nepalganj | 2006-2007 | CT | 112 | 15 (13.4) |
| [91] Shariq 2007 (cited) | Nepalganj? | 2002? | CT | 50 | 25 (50.0) |
| [92] Shrestha 2008 | Lumbini | 2003 | CT, MRI | 93 | 68 (73.1) |
| [93] Gauchan 2011 | Pokhara | 2004-2009 | CT | 678 | 109 (16.1) |
| [94] Thapa 2012 | Chitwan | 2009 | CT | 20 | 4 (20.0) |
| [95] Sapkota 2005* (admission episodes) | Kathmandu | 2000-2004 | NA | 1572 | 294 (18.7) |
| [95] Sapkota 2005* (OPD) | Lalitpur | 2000-2004 | NA | 1717 | 742 (43.2) |
| [96] Pandey 2007* (admission episodes) | Kathmandu, Chitwan | 2002-2006 | NA | 1417 | 189 (13.3) |
| [96] Pandey 2007* (OPD) | Chitwan | 2002-2006 | NA | 2058 | 652 (31.7) |
| [97] Shakya 2009* | Kathmandu | 2003-2008 | NA | 708 | 98 (13.4) |

* Based on hospital registers; excluded from meta-analysis as it cannot be ascertained whether numerator and denominator data correspond to each other

The epilepsy case fatality ratio (i.e., the proportion of epilepsy patients dying per year due to epilepsy) was obtained from the Annual Reports of the Ministry of Health and Population's Department of Health Services (<http://dohs.gov.np/>). A Uniform distribution was modeled based on the lowest and highest case fatality ratio in the period 2001-2011.

Table S3-6. Neurocysticercosis DALY parameters

| **Parameter** | **Distribution** | **Value (95% Range*)** | **Reference** |
| --- | --- | --- | --- |
| Epilepsy prevalence | Uniform(0.0025, 0.0073) | 0.0049 (0.0026–0.0072) | *See text* |
| Mean duration of epilepsy (years) | Fixed | 3.68 | [85] |
| Proportion of epilepsy cases associated with NCC | Beta(5.542, 13.251) | 0.295 (0.118–0.513) | *See text* |
| Epilepsy case fatality ratio | Uniform(0.0081, 0.0226) | 0.0154 (0.0085– 0.0222) | *See text* |
| Proportion of epilepsy patients receiving proper treatment | Fixed | 0.20 | [87] |
| Disability Weight for untreated epilepsy in patients younger than 5 | Beta(3, 27.3) | 0.099 (0.022–0.226) | [85] |
| Disability Weight for untreated epilepsy in patients aged 5 or older | Beta(3, 17) | 0.150 (0.034–0.331) | [85] |
| Disability Weight for treated epilepsy in patients aged 5 or older | Beta(1.5, 35) | 0.041 (0.003–0.124) | [85] |
| Disability Weight for treated epilepsy in patients older than 5 | Beta(1.5, 21.6) | 0.065 (0.005–0.193) | [85] |
| Duration of epilepsy in males younger than 5 (years) | Fixed | 1.4 | [85] |
| Duration of epilepsy in females younger than 5 (years) | Fixed | 1.6 | [85] |
| Duration of epilepsy in males aged 5-14 (years) | Fixed | 2.0 | [85] |
| Duration of epilepsy in females aged 5-14 (years) | Fixed | 3.1 | [85] |
| Duration of epilepsy in males aged 15-44 (years) | Fixed | 3.6 | [85] |
| Duration of epilepsy in females aged 15-44 (years) | Fixed | 5.9 | [85] |
| Duration of epilepsy in males aged 45-59 (years) | Fixed | 2.8 | [85] |
| Duration of epilepsy in females aged 45-59 (years) | Fixed | 6.0 | [85] |
| Duration of epilepsy in males aged 60 or older (years) | Fixed | 1.6 | [85] |
| Duration of epilepsy in females aged 60 or older (years) | Fixed | 2.8 | [85] |

* Defined as the 2.5^th^ and 97.5^th^ percentile of the corresponding distribution

# 5. Toxoplasmosis

The burden assessment for congenital toxoplasmosis is based on Havelaar et al. [98] and Kortbeek et al. [99]. Given the lack of data on the clinical characteristics of congenital toxoplasmosis in Nepal, we assumed these characteristics to be similar as in the Netherlands. Figure S3-2 shows the underlying computational disease model. Table S3-8 summarizes the applied DALY parameters.

Congenital Toxoplasmosis

incidence

Chorioretinitis at birth

Chorioretinitis

later in life

Intracranial

calcifications

CNS abnormalities

Hydrocephalus

Neonatal death

Fetal death

Figure S3-2. Computational disease model for congenital toxoplasmosis. Rectangles with sharp corners represent incidences, those with rounded corners conditional probabilities. Green shapes contribute YLDs, red shapes contribute YLLs.

To estimate the incidence of congenital toxoplasmosis, we followed the hierarchical model presented by Havelaar et al. [98]:

*Incidence toxoplasmosis * (9/12) * P(mother is seronegative) * P(transfer to foetus)*

This model was implemented in a Bayesian framework, allowing to flexibly incorporate uncertainty in the different steps (Model S3-1). First, the incidence of toxoplasmosis is estimated from age-specific IgG seroprevalence data (Table S3-7; [100]), assuming a time homogenous disease transmission model with constant force of infection. The applied seroprevalence dataset was based on a sample of 155 patients visiting Om Hospital and Research Centre, Kathmandu, between 2009 and 2010. The probability that a mother at a certain age is seronegative, is then modeled from the resulting incidence and the age distribution of pregnancies in Nepal. The latter was based on DHS 2006 age-specific fertility data [101], and modeled as a Beta-PERT distribution, with minimum 15, maximum 49, and most likely 28. Finally, the probability that, given a primo-infection of the mother, transfer to the fetus takes place, was modeled based on Thiebaut et al. [102]. Sensitivity analyses showed that the model estimates were robust against alternative prior specifications.

Table S3-7. Age-specific *Toxoplasma* IgG seroprevalence

| **Age Group (Mean)** | **Sample Size** | **Number Positive (%)** |
| --- | --- | --- |
| 0-2 (1) | 4 | 0 (0.0) |
| 11-20 (15) | 5 | 1 (20.0) |
| 21-30 (25) | 112 | 31 (27.7) |
| 31-40 (35) | 29 | 9 (31.0) |
| 41-50 (45) | 5 | 0 (0.0) |

Model S3-1. Bayesian estimation of the congenital toxoplasmosis incidence based on age-specific seroprevalence data

model {

congtp <- 1000 * (1 - prevalence) * incidence * (9/12) * p.transfer

prevalence <- 1 - pow((1 - incidence), age)

age <- age.beta * (49 - 15) + 15

age.beta ~ dbeta(2.868, 4.633)

p.transfer ~ dbeta(506, 1215)

for (i in 1:N){

p[i] ~ dbin(prev[i], n[i])

prev[i] <- max(min(1 - pow((1 - incidence), a[i]), 1), 0)

}

incidence ~ dgamma(1, 100)

}

Table S3-8. Congenital toxoplasmosis DALY parameters

| **Parameter** | **Distribution** | **Value (95% Range*)** | **Reference** |
| --- | --- | --- | --- |
| Incidence congenital toxoplasmosis (per 1000 live births)** | Gamma(57.223, 31.354) | 1.825 (1.383–2.327) | *See text* |
| Number of births, 2005 | Fixed | 764,700 | [103] |
| Proportion of infected neonates with chorioretinitis at birth | Beta(141, 907) | 0.13 (0.11–0.16) | [98] |
| Proportion of infected neonates with chorioretinitis later in life | Uniform(0.086, 0.237) | 0.16 (0.09–0.23) | [98] |
| Proportion of infected neonates with hydrocephalus | Beta(16, 840) | 0.02 (0.01–0.03) | [98] |
| Proportion of infected neonates with intracranial calcifications | Beta(88, 749) | 0.11 (0.09–0.13) | [98] |
| Proportion of infected neonates with CNS abnormalities | Beta(3, 102) | 0.03 (0.01–0.07) | [98] |
| Proportion of infected neonates that die | Beta(9, 1202) | 0.01 (0–0.01) | [98] |
| Number of fetal deaths per infected neonate | BetaPERT(0.01, 0.03, 0.09) | 0.04 (0.02–0.06) | [98] |
| Disability Weight for patients with chorioretinitis | Fixed | 0.08 | [98] |
| Disability Weight for patients with hydrocephalus | Fixed | 0.36 | [98] |
| Disability Weight for patients with intracranial calcifications | Fixed | 0.01 | [98] |
| Disability Weight for patients with CNS abnormalities | Fixed | 0.36 | [98] |
| Duration for patients with chorioretinitis (years) | Fixed | *Lifelong* | [98] |
| Duration for patients with hydrocephalus (years) | Fixed | *Lifelong* | [98] |
| Duration for patients with intracranial calcifications (years) | Fixed | *Lifelong* | [98] |
| Duration for patients with CNS abnormalities (years) | Fixed | *Lifelong* | [98] |

* Defined as the 2.5^th^ and 97.5^th^ percentile of the corresponding distribution

** Note that this parameter corresponds to the incidence of clinical and non-clinical congenital toxoplasmosis; the value in Table 6 corresponds to the estimated incidence of clinical cases only.

# 6. Cystic echinococcosis

The burden assessment for cystic echinococcosis (CE) is based on Budke et al. [104]. Figure S3-3 shows the underlying computational disease model. Table S3-11 summarizes the applied DALY parameters.

Surgical CE

incidence

Recovery

Substantial post-surgical conditions

Post-surgical death

*Non-reported cases*

Recurrent disease

Figure S3-3. Computational disease model for cystic echinococcosis. Rectangles with sharp corners represent incidences, those with rounded corners conditional probabilities or ratios (indicated in italics). Green shapes contribute YLDs, red shapes contribute YLLs.

To assess the annual incidence of surgical CE cases, we estimated the annual number of CE surgeries by reviewing the surgical ward registers of the major hospitals of Nepal (Table S3-9). As CE surgery comes with the risk of anaphylaxis in case of cyst rupture, this surgery is generally performed in well-equipped hospitals, which typically have 100+ beds. In Nepal, the major hospitals are centered in four areas, i.e., the Kathmandu Valley, the Pokhara Valley (Kaski), the Biratnagar-Dharan axis (Sunsari, Morang), and the Bharatpur area (Chitwan). The number of cases per bed further appears to depend on the price of the intervention and on the reputation of the hospital and its surgeons. The total number of annual cases was found to be 89, which corresponds to an annual incidence of 89/26,271,653 or 0.34 per 100,000 per year. To account for the uncertainty in this estimate due to the incomplete coverage of our hospital survey, we applied it as the minimum value in a Uniform distribution, of which the maximum value was arbitrarily set equal to two times this estimate. Following Budke et al. [104], we also calculated the number of unreported and untreated cases, based on the assumption that ~10% of all cases remains untreated and thus unreported. In other words, the ratio of untreated versus treated cases is 1:9, or 0.11.

Table S3-9. Estimated annual number of surgical Cystic Echinococcosis (CE) cases in the major Nepalese hospitals

| **Hospital name*** | **Location (District)** | **# beds** | **#CE/y** |
| --- | --- | --- | --- |
| Bharatpur Hospital | Bharatpur (Chitwan) | 270 | 5 |
| Chitwan Medical College TH | Bharatpur (Chitwan) | 500 | 7 |
| College of Medical Sciences TH | Bharatpur (Chitwan) | 1050 | 7 |
| Gandaki Medical College TH | Pokhara (Kaski) | 300 | 7 |
| Gandaki Regional Hospital | Pokhara (Kaski) | 300 | 10 |
| Manipal College of Medical Sciences TH | Pokhara (Kaski) | 825 | 8 |
| Bir Hospital | Kathmandu (Kathmandu) | 535 | 10 |
| Kanti Children Hospital | Kathmandu (Kathmandu) | 300 | 1 |
| Kathmandu Model Hospital | Kathmandu (Kathmandu) | 125 | 4 |
| Tribhuvan University TH | Kathmandu (Kathmandu) | 450 | 15 |
| Patan Hospital | Lalitpur (Lalitpur) | 450 | 5 |
| BP Koirala Institute of Health Sciences | Dharan (Morang) | 700 | 10 |

*TH = Teaching Hospital

In addition, we estimated the age and sex distribution of treated CE cases from hospital registers (based on Gautam [105], Bashyal [106], and own data collection) and the register of a private medical laboratory. In total, this yielded data on 238 CE patients (Table S3-10).

Table S3-10. Age and sex distribution of 238 CE patients. Count (Percentage of total).

| **Age** | **Sex** |  |  |
| --- | --- | --- | --- |
|  | Male | Female | All |
| **0-4** | 4 (1.7%) | 3 (1.3%) | 7 (2.9%) |
| **5-14** | 13 (5.5%) | 6 (2.5%) | 19 (8.0%) |
| **15-44** | 49 (20.6%) | 83 (34.9%) | 132 (55.5%) |
| **45-59** | 20 (8.4%) | 32 (13.4%) | 52 (21.8%) |
| **60+** | 13 (5.5%) | 15 (6.3%) | 28 (11.8%) |
| **Total** | **99 (41.6%)** | **139 (58.4%)** | **238 (100%)** |

Table S3-11. Cystic echinococcosis DALY parameters (based on Budke et al. [104])

| **Parameter** | **Distribution** | **Value (95% Range*)** |
| --- | --- | --- |
| Proportion of patients who recover after surgery | Dirichlet({533, 118, 46, 16}) | 0.75 (0.72–0.78) |
| Proportion of patients who experience substantial postsurgical conditions |  | 0.17 (0.14–0.19) |
| Proportion of patients who experience recurrent disease |  | 0.06 (0.05–0.08) |
| Proportion of patients who die after surgery |  | 0.02 (0.01–0.03) |
| Ratio of untreated versus treated cases | Fixed | 0.11 |
| Disability Weight for patients who recover | Fixed | 0.200 |
| Disability Weight for patients who experience substantial postsurgical conditions | Fixed | 0.239 |
| Disability Weight for patients who experience recurrent disease | Fixed | 0.809 |
| Disability Weight for patients who remain untreated | Fixed | 0.200 |
| Duration for patients that recover (years) | Fixed | 1 |
| Duration for patients with substantial postsurgical conditions (years) | Fixed | 5 |
| Duration for patients with recurrent disease (years) | Fixed | 5 |
| Duration for patients who remain untreated (years) | Fixed | 10 |

* Defined as the 2.5^th^ and 97.5^th^ percentile of the corresponding distribution

# References

1. Gaihre YK (2000) Prevalence of intestinal helminth parasites in general, ascariasis in detail in Sarkies and Magars community of Tindobate VDC, Syangja, Nepal [Msc dissertation]. Kathmandu: Tribhuvan University. 92 p.

2. Thapa RB (2000) Prevalence of intestinal helminth parasites in general and *Taenia* spp in detail, particularly in Bote and Darai communities of Vyash Muncipality-5, Kumaltari, Tanahun district of Nepal [MSc dissertation]. Kathmandu: Tribhuvan University. 91 p.

3. Pradhan SK (2001) Prevalence of intestinal helminth parasitosis in Magar and Majhi children of PragataNagar VDC of District Nawalparasi: A community-based study [MSc dissertation]. Kathmandu: Tribhuvan University. 52 p.

4. Karki D (2003) An epidemiological survey on intestinal helminthes among Magar communties in Barangdi VDC, Palpa, with special reference to *Taenia* spp. [MSc dissertation]. Kathmandu: Tribhuvan University. 56 p.

5. Karki S (2003) Prevalence of intestinal parasites in humans and dogs of KMC particularly in Ward no 20, KMC [MSc dissertation]. Kathmandu: Tribhuvan University. 69 p.

6. Parajuli RP (2003) Prevalence of intestinal parasitosis in Mushar Community of Chitwan district in relation to their socio-cultural and socio-economic status [MSc dissertation]. Kathmandu: Tribhuvan University. 146 p.

7. Sharma P (2007) Study on intestinal parasitic infections in Tharu community of Bardiya district [MSc dissertation]. Kathmandu: Tribhuvan University.

8. Shrestha A (2007) Prevalence of soil transmitted parasites in raw vegetables of Kathmandu Valley and stool samples of school children [MSc dissertation]. Kathmandu: Tribhuvan University.

9. Devleesschauwer B, Aryal A, Joshi DD, Rijal S, Sherchand JB, et al. (2012) Epidemiology of *Taenia solium* in Nepal: is it influenced by the social characteristics of the population and the presence of *Taenia asiatica*? Trop Med Int Health 17: 1019-1022.

10. Gyawali P (2012) Parasitic diseases of indigenous community (Kumal) in Nepal. Nepal Journal of Science and Technology 13: 175-178.

11. Gyawali PK (2003) Baseline study on gastrointestinal parasitic infection of Kumal at Gaindakot VDC, Nawalparasi district [MSc dissertation]. Kathmandu: Tribhuvan University. 52 p.

12. Lee IL, Tan TC, Tan PC, Nanthiney DR, Biraj MK, et al. (2012) Predominance of *Blastocystis* sp. subtype 4 in rural communities, Nepal. Parasitol Res 110: 1553-1562.

13. Sah RB, Pokharel PK, Paudel IS, Acharya A, Jha N, et al. (2012) A study of prevalence of *Taenia* infestation and associated risk factors among the school children of dharan. Kathmandu Univ Med J (KUMJ) 10: 14-17.

14. Joshi DD, Poudyal MP, Jimba M, Mishra PN, Neave L, et al. (2001) Epidemiological status of *Taenia*/cysticercosis in pigs and human in Nepal. Journal of the Institute of Medicine 23: 12.

15. Das R, Pradipta Swain K, Biswas R (2006) Prevalence of intestinal parasites and its association with sociodemographic, environmental and behavioral factors in children in Pokhara valley, Nepal. African Journal of Clinical and Experimental Microbiology 7: 106-115.

16. Sherchand JB (2006) Hospital and clinic based study on taeniasis in Nepal. In: Joshi DD, Sharma M, Rana S, editors. Present situation challenges in treatment and elimination of taeniasis/cysticercosis in Nepal. Kathmandu, Nepal: National Zoonoses and Food Hygiene Research Centre. pp. 69-73.

17. Maharjan B (2009) Prevalence of intestinal parasites in children attending OPD of Kanti Children Hospital, Kathmandu [MSc dissertation]. Kathmandu: Tribhuvan University. 54 p.

18. Shakya B, Bhargava D, Shrestha S, Rijal BP (2009) Intestinal parasitosis. Journal of Institute of Medicine 31: 13-16.

19. Khanal LK, Rai SK, Khanal PR, Ghimire G (2011) Status of intestinal parasitosis among hospital visiting patients in Deukhury Valley, Dang, Nepal. Nepal Med Coll J 13: 100-102.

20. Thapa B, Salhotra VS, Jha KK (2011) Temporal distribution of intestinal parasites in Kathmandu, Nepal. SAARC TB and HIV/AIDS Centre Newsletter 21: 15-17.

21. Ghimire K (2002) Prevalence of intestinal parasites in humans and dogs of KMC particularly in Ward no 19, KMC [MSc dissertation]. Kathmandu: Tribhuvan University. 68 p.

22. Shrestha R (2000) Prevalence of intestinal parasites upto 10 years old children in Kathmandu and Bhaktapur [MSc dissertation]. Kathmandu: Tribhuvan University. 74 p.

23. Yong TS, Sim S, Lee J, Ohrr H, Kim MH, et al. (2000) A small-scale survey on the status of intestinal parasite infections in rural villages in Nepal. Korean J Parasitol 38: 275-277.

24. Rijal B, Oda Y, Basnet R, Parajuli K, Gurung CK, et al. (2001) Gender variations in the prevalence of parasitic infections and the level of awareness in adolescents in rural Nepal. Southeast Asian J Trop Med Public Health 32: 575-580.

25. Goto R, Panter-Brick C, Northrop-Clewes CA, Manahdhar R, Tuladhar NR (2002) Poor intestinal permeability in mildly stunted Nepali children: associations with weaning practices and *Giardia lamblia* infection. Br J Nutr 88: 141-149.

26. Williams-Blangero S, VandeBerg JL, Subedi J, Aivaliotis MJ, Rai DR, et al. (2002) Genes on chromosomes 1 and 13 have significant effects on *Ascaris* infection. Proceedings of the National Academy of Sciences 99: 5533-5538.

27. Chaudhary B (2003) Hospital and community based study on infection of human intestinal parasites in relation to sociomedical aspects [MSc dissertation]. Kathmandu: Tribhuvan University. 73 p.

28. Moffat T (2003) Diarrhea, respiratory infections, protozoan gastrointestinal parasites, and child growth in Kathmandu, Nepal. Am J Phys Anthropol 122: 85-97.

29. Rai DR (2003) Study of the factors associated with enteric parasitic infection among school children in a rural village setting in Kathmandu Valley, Nepal [MSc dissertation]. Kathmandu: Tribhuvan University. 61 p.

30. Sherchand J, Cross JH (2003) *Cyclospora cayetanensis* in Nepal: a study of microbiological and epidemiological aspects. J Nepal Health Res Counc 3: 1-8.

31. Malla B, Sherchan JB, Ghimire P, Rajendra Kumar BC, Gauchan P (2004) Prevalence of intestinal parasitic infections and malnutrition among children in a rural community of Sarlahi, Nepal. J Nepal Health Res Counc 2: 4.

32. Sharma BK, Rai SK, Rai DR, Choudhury DR (2004) Prevalence of intestinal parasitic infestation in schoolchildren in the northeastern part of Kathmandu Valley, Nepal. Southeast Asian J Trop Med Public Health 35: 501-505.

33. Williams-Blangero S, Correa-Oliveira R, Vandeberg JL, Subedi J, Upadhayay RP, et al. (2004) Genetic influences on plasma cytokine variation in a parasitized population. Hum Biol 76: 515-525.

34. Ghimire TR, Mishra PN (2005) Intestinal parasites and haemoglobin concentration in the people of two different areas of Nepal. J Nepal Health Res Counc 3: 1-7.

35. Kunwar C, Chapagain R, Subba B, Shrestha M, Jha B, et al. (2006) Occurrence of soil-transmitted helminths in women at the Himalayan region of Nepal. Kathmandu Univ Med J (KUMJ) 4: 444-447.

36. Majhi Tharu HN (2006) Epidemiology of intestinal parasites among Chepang adults at Taklung VDC of Gorkha [MSc dissertation]. Kathmandu: Tribhuvan University. 62 p.

37. Shakya B, Rai SK, Singh A, Shrestha A (2006) Intestinal parasitosis among the elderly people in Kathmandu Valley. Nepal Med Coll J 8: 243-247.

38. Adhikari N, Bomjan R, Khatri D, Joshi D, Dhakal P, et al. (2007) Intestinal helminthic infections among school children in Kathmandu valley. J Nepal Health Res Counc 5: 17-21.

39. Albonico M, Mathema P, Montresor A, Khakurel B, Reggi V, et al. (2007) Comparative study of the quality and efficacy of originator and generic albendazole for mass treatment of soil-transmitted nematode infections in Nepal. Trans R Soc Trop Med Hyg 101: 454-460.

40. Jamarkattel MP, Ghimire TR (2007) Intestinal parasitic infection with relation to socio-economic status of Jalari and Kumal communities in Lekhnath Municipality, Kaski, Nepal. J Nepal Health Res Counc 5: 13-20.

41. Shrestha A, Rai SK, Basnyat SR, Rai CK, Shakya B (2007) Soil transmitted helminthiasis in Kathmandu, Nepal. Nepal Med Coll J 9: 166-169.

42. Rai S, Gurung R, Saiju R, Bajracharya L, Rai N, et al. (2008) Intestinal parasitosis among subjects undergoing cataract surgery at the eye camps in rural hilly areas of Nepal. Nepal Med Coll J 10: 100-103.

43. Albonico M, Allen H, Mathema P, Giri D, Shrestha R, et al. (2009) Monitoring albendazole efficacy after regular treatment of soil-transmitted helminth infections in Nepali children. 6th European Congress on Tropical Medicine and International Health. Verona, Italy: Tropical Medicine and International Health. pp. 55-56.

44. Gyawali N, Amatya R, Nepal HP (2009) Intestinal parasitosis in school going children of Dharan municipality, Nepal. Trop Gastroenterol 30: 145-147.

45. Parajuli RP, Umezaki M, Watanabe C (2009) Behavioral and nutritional factors and geohelminth infection among two ethnic groups in the Terai region, Nepal. American Journal of Human Biology 21: 98-104.

46. Bhandari N, Kausaph V, Neupane G (2011) Intestinal parasitic infection among school age children. J Nepal Health Res Counc 9: 30-32.

47. Thapa Magar D, Rai SK, Lekhak B, Rai KR (2011) Study of parasitic infection among children of Sukumbasi Basti in Kathmandu valley. Nepal Med Coll J 13: 7-10.

48. Shakya B, Shrestha S, Madhikarmi N, Adhikari R (2012) Intestinal parasitic infection among school children. J Nepal Health Res Counc 10: 20-23.

49. Shrestha A, Narayan K, Sharma R (2012) Prevalence of intestinal parasitosis among school children in Baglung District of Western Nepal. Kathmandu Univ Med J (KUMJ) 37: 3-7.

50. Chand AB (2000) A prospective study on aetiological agents of diarrhoeal disease in children in relation to parasites and to determine the antibiotic sensitivity pattern of bacterial isolates [MSc dissertation]. Kathmandu: Tribhuvan University. 74 p.

51. Shrestha AK, Enriquez FJ (2001) Short report: Prevalence of fecal *Encephalitozoon* sp. spores among hospitalized patients in Nepal. Am J Trop Med Hyg 65: 648-649.

52. Pandey BD, Thapa LB, Sherchand JB, Rimal N, Bhattarai A, et al. (2002) Etiology of diarrhoea among adult patients during the early monsoon period in Kathmandu, Nepal. Japanese Journal of Tropical Medicine and Hygiene 30: 133-137.

53. Rai K, Sherchand JB, Bhatta DR (2004) Study of enteropathogens and its predisposing factors in gastroenteritis suspected children attending Kanti Children Hospital, Kathmandu, Nepal. J Nepal Assos Med Lab Sciences 6: 48-53.

54. Uga S, Rai SK, Kimura K, Ganesh R, Kimura D, et al. (2004) Parasites detected from diarrheal stool samples collected in Nepal. Southeast Asian J Trop Med Public Health 35: 19-23.

55. Khadka D (2005) Detection of enteropathogens (*Salmonella* spp, *Shigella* spp and parasites) in the stool specimen of children suffering from diarrhoea and admitted at Kanti Children Hospital [MSc dissertation]. Kathmandu: Tribhuvan University. 89 p.

56. Lama C (2006) Microbiological study among diarrhoeal children in relation to *Cyclospora* and rotavirus infection [MSc dissertation]. Kathmandu: Tribhuvan University.

57. Lama C, Sherchan JB (2007) Enteropathogens associated diarrhea in hospitalized patients of Children’s Hospital, Kathmandu. J Nepal Health Res Counc 5: 50-57.

58. Sherchand JB, Yokoo M, Sherchand O, Pant AR, Nakogomi O (2009) Burden of enteropathogens associated diarrheal diseases in children hospital, Nepal. Scientific World 7: 71-75.

59. Mukhopadhyay C, Wilson G, Pradhan D, Shivananda PG (2007) Intestinal protozoal infestation profile in persistent diarrhea in children below age 5 years in western Nepal. Southeast Asian J Trop Med Public Health 38: 13-19.

60. Kandel S (2008) Intestinal parasitic infection in children in Kathmandu Valley, Nepal: a study in Kanti children's hospital, Maharajgunj, Kathmandu, Nepal [MSc dissertation]. Kathmandu: Tribhuvan University. 71 p.

61. Shrestha SD, Malla S, Basnyat SR (2008) Etiology of diarrhoea with reference to multiple drug resistant enteric bacterial pathogens. Nepal Journal of Science and Technology 9: 131-138.

62. Tandukar S, Sherchand O, Singh A, Sherchand J (2008) Enteropathogenic microorganisms in children. Journal of Institute of Medicine 30: 17-22.

63. Pokharel M, Sherchand J, Upreti H, Katuwal A, Gauchan P (2009) A perspective study on the etiology of diarrhea in children less than 12 years of age attending Kanti Children’s Hospital. Journal of Nepal Paediatric Society 29: 10-16.

64. Basnet A, Sherchan J, Rijal B, Sharma S, Khadga P (2010) Detection of coccidian parasites and their clinical manifestation, treatment and prophylaxis in HIV infected patients in Tribhuvan University Teaching Hospital. Scientific World 8: 51-55.

65. Amatya R, Poudyal N, Gurung R, Khanal B (2011) Prevalence of *Cryptosporidium* species in paediatric patients in Eastern Nepal. Trop Doct 41: 36-37.

66. Ansari S, Sherchand JB, Parajuli K, Paudyal BM, Adhikari RP, et al. (2012) Pattern of acute parasitic diarrhea in children under five years of age in Kathmandu, Nepal. Open Journal of Medical Microbiology 2: 95-100.

67. Sapkota D (2003) Prevalence of enteric parasitosis in HIV/aids patients of Nepal [MSc dissertation]. Kathmandu: Tribhuvan University. 80 p.

68. Sapkota D, Ghimire P, Manandhar S (2004) Enteric parasitosis in patients with human immunodeficiency virus (HIV) Infection and acquired immunodeficiency syndrome (AIDS) in Nepal. J Nepal Health Res Counc 2: 9-13.

69. Adhikari N (2006) Study of prevalence of intestinal parasitic infection among HIV seropositive subjects and high risk group for HIV infection in Bagmati Zone, Nepal [MSc dissertation]. Kathmandu: Tribhuvan University. 86 p.

70. Amatya R, Shrestha R, Poudyal N, Bhandari S (2011) Opportunistic intestinal parasites and CD4 count in HIV infected people. Journal of Pathology of Nepal 1: 118-121.

71. Shariff M, Deb M, Singh R, Singh K (2002) *Cryptosporidium* infection in children with diarrhoea of acute onset. J Trop Pediatr 48: 187-188.

72. Ghimire T, Mishra P, Sherchand J (2005) The seasonal outbreaks of *Cyclospora* and *Cryptosporidium* in Kathmandu, Nepal. J Nepal Health Res Counc 3: 39-48.

73. Majhi Tharu HN (2006) Epidemiology of intestinal parasites among Chepang adults at Taklung VDC of Gorkha [MSc dissertation]. Kathmandu: Tribhuvan University. 62 p.

74. Ono K, Rai SK, Chikahira M, Fujimoto T, Shibata H, et al. (2001) Seasonal distribution of enteropathogens detected from diarrheal stool and water samples collected in Kathmandu, Nepal. Southeast Asian J Trop Med Public Health 32: 520-526.

75. Rai K, Sherchand JB, Bhatta DR, Bhattarai NR (2005) Status of *Giardia intestinalis* infection among the children attending Kanti Children Hospital, Nepal. Scientific World 3: 102-105.

76. Dhakal DN, Rajendra Kumar BC, Sherchand JB, Mishra PN (2004) *Cryptosporidium parvum*: An Observational Study in Kanti Children Hospital, Kathmandu, Nepal. J Nepal Health Res Counc 2: 1-5.

77. Singh A, Janaki L, Petri WA, Jr., Houpt ER (2009) *Giardia intestinalis* assemblages A and B infections in Nepal. Am J Trop Med Hyg 81: 538-539.

78. Yoshikawa H, Wu Z, Pandey K, Pandey BD, Sherchand JB, et al. (2009) Molecular characterization of *Blastocystis* isolates from children and rhesus monkeys in Kathmandu, Nepal. Vet Parasitol 160: 295-300.

79. Sherchand J, Tandukar S, Sherchan J, Rayamajhi A, Gurung B, et al. (2012) Hospital-based study in children with rotavirus gastroenteritis and other enteropathogens. J Nepal Health Res Counc 10: 130-135.

80. Ghimire P, Sapkota D, Manandhar SP (2004) Cryptosporidiosis: opportunistic infection in HIV/AIDS patients in Nepal. J Trop Med Parasitol 27: 7-10.

81. Das R, Joshi H, Biswas R (2005) Opportunistic infections and clinico-epidemiological factors in HIV/AIDS cases seen in a tertiary care hospital in Nepal. African Journal of Clinical and Experimental Microbiology 6: 239-245.

82. Mishra B, Sinha ND, Shukla S, Das R (2009) The epidemiology of opportunistic infections in HIV/AIDS cases in Nepal. Indian J Prev Soc Med 40: 97-100.

83. Sharma S, Dhungana G, Pokhrel B, Rijal B (2010) Opportunistic infections in relation to CD4 level among HIV seropositive patients from central Nepal. Nepal Med Coll J 12: 1-4.

84. Sherchan J, Ohara H, Sakurada S, Basnet A, Tandukar S, et al. (2012) Enteric opportunistic parasitic infections among HIV-seropositive patients in Kathmandu, Nepal. Kathmandu Univ Med J (KUMJ) 38: 14-17.

85. Praet N, Speybroeck N, Manzanedo R, Berkvens D, Nsame Nforninwe D, et al. (2009) The disease burden of *Taenia solium* cysticercosis in Cameroon. PLoS Negl Trop Dis 3: e406.

86. UNICEF (2001) A situation analysis of disability in Nepal. Kathmandu, Nepal: United Nations Children’s Fund, Nepal and His Majesty’s Government, Nepal, National Planning Commission. 307 p.

87. Rajbhandari KC (2004) Epilepsy in Nepal. Can J Neurol Sci 31: 257-260.

88. Neupane A (2006) Neurocysticercosis in children admitted in Birendra Hospital Chauni, Kathmandu. In: Joshi DD, Sharma M, Rana S, editors. Present situation challenges in treatment and elimination of taeniasis/cysticercosis in Nepal. Kathmandu, Nepal: National Zoonoses and Food Hygiene Research Centre. pp. 148-154.

89. Chaudhary S (2006) Present situation of neurocysticercosis in Patan hospital. In: Joshi DD, Sharma M, Rana S, editors. Present situation challenges in treatment and elimination of taeniasis/cysticercosis in Nepal. Kathmandu, Nepal: National Zoonoses and Food Hygiene Research Centre. pp. 155-162.

90. Piryani R, Kohli S, Shrestha G, Shukla A, Malla T (2007) Human neurocysticercosis managed at Nepalganj Medical College, Teaching Hospital, Kohalpur, Nepal. Kathmandu Univ Med J (KUMJ) 20: 518-520.

91. Shariq SM, Adhikari BP (2007) Managing cysticercosis in anterior chamber of eye: a case report. Kathmandu Univ Med J (KUMJ) 5: 240-242.

92. Shrestha B (2008) Childhood neurocysticercosis: Clinico-radiological profile and outcome. Journal of Nepal Paediatric Society 28: 14-16.

93. Gauchan E, Malla T, Basnet S, Rao KS (2011) Variability of presentations and CT-scan findings in children with neurocysticercosis. Kathmandu Univ Med J (KUMJ) 9: 17-21.

94. Thapa L, Shrestha A, Paudel R, Pokharel B, Ghimire A, et al. (2012) Clinical and socio-economic factors among epileptic patients in Nepal: a big challenge. Journal of College of Medical Sciences-Nepal 7: 29-33.

95. Sapkota BS (2005) Prevalence of porcine cysticercosis and trichinellosis in slaughter pigs in Kathmandu Valley, Nepal [MSc dissertation]. Chiang Mai and Berlin: Chiang Mai University and Freie Universität Berlin. 75 p.

96. Pandey KR (2007) Prevalence and comparision of carcass examination, lingual examination and EITB for the diagnosis of porcine cysticercosis in Nepal [MSc dissertation]. Rampur: Tribhuvan University, Institute of Agriculture and Animal Science. 93 p.

97. Shakya M (2009) *Taenia solium* in pigs and its public health importance in Kirtipur Municipality [MSc dissertation]. Kathmandu: Tribhuvan University. 48 p.

98. Havelaar AH, Kemmeren JM, Kortbeek LM (2007) Disease burden of congenital toxoplasmosis. Clin Infect Dis 44: 1467-1474.

99. Kortbeek LM, Hofhuis A, Nijhuis CD, Havelaar AH (2009) Congenital toxoplasmosis and DALYs in the Netherlands. Mem Inst Oswaldo Cruz 104: 370-373.

100. Ray R (2010) Seroprevalence of IgM and IgG antibodies against the agents of TORCH infections among the patients visiting om hospital and research center [MSc dissertation]. Kathmandu: Tribhuvan University. 64 p.

101. Ministry of Health and Population (MOHP) [Nepal], New ERA, Macro International Inc (2007) Nepal Demographic and Health Survey 2006. Kathmandu: Ministry of Health and Population, New ERA, and Macro International Inc. 291 p. Available: http://www.measuredhs.com/pubs/pdf/FR191/FR191.pdf. Accessed 12 July 2013.

102. Thiebaut R, Leproust S, Chene G, Gilbert R (2007) Effectiveness of prenatal treatment for congenital toxoplasmosis: a meta-analysis of individual patients' data. Lancet 369: 115-122.

103. United Nations (2010) World Population Prospects: The 2010 Revision. Population Division, Department of Economic and Social Affairs, United Nations New York, NY, USA.

104. Budke CM, Deplazes P, Torgerson PR (2006) Global socioeconomic impact of cystic echinococcosis. Emerg Infect Dis 12: 296-303.

105. Gautam BP (2009) Prevalence of cystic echinococcosis/hydatidosis in slaughtered buffaloes of Kathmandu metropolitan city and assessment of its impact on public health [BSc dissertation]. Rampur: Tribhuvan University, Institute of Agriculture and Animal Sciences. 55 p.

106. Bashyal K (2003) Case study of echinococcosis/hydatidosis in different hospitals of Kathmandu [MSc dissertation]. Kathmandu: Tribhuvan University. 60 p.
